# Kaposi’s sarcoma-associated herpesvirus vIRF2 protein utilizes an IFN-dependent pathway to regulate viral early gene expression

**DOI:** 10.1371/journal.ppat.1007743

**Published:** 2019-05-06

**Authors:** Sandra Koch, Modester Damas, Anika Freise, Elias Hage, Akshay Dhingra, Jessica Rückert, Antonio Gallo, Elisabeth Kremmer, Werner Tegge, Mark Brönstrup, Wolfram Brune, Thomas F. Schulz

**Affiliations:** 1 Hannover Medical School, Institute of Virology, Hannover, Germany; 2 German Centre for Infection Research, Hannover-Braunschweig Site, Germany; 3 Heinrich-Pette-Institute, Leibniz Institute for Experimental Virology, Hamburg, Germany; 4 German Centre for Infection Research, Hamburg Site, Germany; 5 Institute of Molecular Immunology, Helmholtz Centre Munich, German Research Center for Environmental Health, Munich, Germany; 6 Helmholtz Centre for Infection Research, Braunschweig, Germany; University of North Carolina at Chapel Hill, UNITED STATES

## Abstract

Kaposi’s sarcoma-associated herpesvirus (KSHV; human herpesvirus 8) belongs to the subfamily of *Gammaherpesvirinae* and is the etiological agent of Kaposi’s sarcoma as well as of two lymphoproliferative diseases: primary effusion lymphoma and multicentric Castleman disease. The KSHV life cycle is divided into a latent and a lytic phase and is highly regulated by viral immunomodulatory proteins which control the host antiviral immune response. Among them is a group of proteins with homology to cellular interferon regulatory factors, the viral interferon regulatory factors 1–4. The KSHV vIRFs are known as inhibitors of cellular interferon signaling and are involved in different oncogenic pathways. Here we characterized the role of the second vIRF protein, vIRF2, during the KSHV life cycle. We found the vIRF2 protein to be expressed in different KSHV positive cells with early lytic kinetics. Importantly, we observed that vIRF2 suppresses the expression of viral early lytic genes in both newly infected and reactivated persistently infected endothelial cells. This vIRF2-dependent regulation of the KSHV life cycle might involve the increased expression of cellular interferon-induced genes such as the IFIT proteins 1, 2 and 3, which antagonize the expression of early KSHV lytic proteins. Our findings suggest a model in which the viral protein vIRF2 allows KSHV to harness an IFN-dependent pathway to regulate KSHV early gene expression.

## Author summary

The life cycle of Kaposi Sarcoma herpesvirus involves both persistence in a latent form and productive replication to generate new viral particles. How the virus switches between latency and productive (‘lytic’) replication is only partially understood. Here we show that a viral homologue of interferon regulatory factors, vIRF2, antagonizes lytic protein expression in endothelial cells. It does this by inducing the expression of cellular interferon-regulated genes such as IFIT 1–3, which in turn dampens early viral gene expression. This observation suggests that vIRF2 allows KSHV to harness the interferon pathway to regulate early viral gene expression in endothelial cells.

## Introduction

Kaposi’s sarcoma-associated herpesvirus (KSHV) or human herpesvirus 8 (HHV8) belongs to the genus *Rhadinovirus* within the subfamily of *Gammaherpesvirinae*. It was identified in 1994 when herpesvirus-like DNA sequences were discovered in AIDS-associated Kaposi's sarcoma (KS) tissues [1]. Apart from KS, KSHV is also the cause of two lymphoproliferative diseases, primary effusion lymphoma (PEL) and multicentric Castleman’s disease (MCD) [2, 3]. The KSHV life cycle is divided into two phases: latency and productive (‘lytic’) replication. Early after infection, the viral dsDNA enters the nucleus, is circularized and further chromatinized and maintained as a stable episome within the host [4]. During latency no viral particles are produced and only a few latent proteins are expressed from the so called latency transcript cluster under the control of a constitutively active promoter. These latent proteins function as viral regulators enabling the establishment and maintenance of latency as well as the inhibition of the lytic cycle. Furthermore, they are involved in cell proliferation, survival, differentiation and transformation as well as angiogenesis and the induction of interferon stimulated genes (ISGs) and thereby contribute to KSHV pathogenesis [4–7]. The latent state can be disrupted by lytic reactivation which is characterized by a distinct pattern of gene expression, involving immediate early, early lytic and late lytic transcripts [8]. Lytic cycle activation can be induced by co-factors like hypoxia, oxidative stress and inflammatory cytokines [8–15]. In addition, viral co-infections with human immunodeficiency virus-1 (HIV-1), Herpes simplex virus (HSV) or Human cytomegalovirus (HCMV) are known to induce KSHV reactivation [13, 16–19]. Several chemical compounds, such as histone deacetylase (HDAC) inhibitors like sodium butyrate (SB), or 12-O-tetradecanoylphorbol-13-acetate (TPA) can activate the lytic replication cycle [20, 21]. The immediate early protein, replication and transcription activator (RTA) encoded by ORF50, is a key viral regulator of the KSHV lytic cycle. By trans-activating the expression of other lytic downstream genes as well as its own promoter, RTA is necessary and sufficient to induce the entire viral lytic cycle [22, 23]. Several viral proteins expressed during the early stages of the lytic replication cycle such as vIL6, vGPCR, K1 and K15 contribute to KSHV pathogenesis by promoting proliferation, angiogenesis, invasiveness and may counteract the host antiviral immune response [24, 25].

The KSHV genome also contains four genes with partial sequence homology to cellular interferon regulatory factors (IRFs): an unspliced mRNA transcribed from the viral open reading frame (ORF) K9 encodes viral interferon regulatory factor 1 (vIRF1), while spliced mRNAs generated from ORF K10/K10.1, ORF K10.5/10.6 and ORF K11/K11.1 yield vIRF 4, 3 and 2, respectively [26–29]. Apart from KSHV, the only other viruses known for now to encode similar viral IRFs are the rhadinovirus of rhesus macaques (RRV) and the retroperitoneal fibromatosis herpesvirus of pig-tailed macaques (RFHVMn) [30–32]. In spite of the low degree of protein sequence homology with cellular IRFs and in particular the only partial conservation of five characteristic tryptophan residues found in the N-terminal DNA binding domain (DBD) of cellular IRFs, at least vIRF1 and vIRF2 have been shown to bind to viral or cellular DNA in a similar manner as cellular IRFs [33–38]. Most studies on the function of individual KSHV vIRFs suggest that they counteract the cellular interferon (IFN) response and inhibit different proliferative, apoptotic and angiogenetic pathways by interacting with and functionally modulating cellular proteins [39]. So far, three vIRFs have been implicated in the regulation of viral lytic replication. Thus, vIRF1 has been shown to promote, and vIRF3 to suppress, KSHV lytic replication by recruitment of USP7 [40]. Interestingly, vIRF3 is expressed as a latent protein only in PEL cells and in MCD tissue, it is important for PEL cell survival and its expression is unchanged after lytic reactivation [41]. Furthermore, vIRF4 has been shown to support lytic reactivation by virtue of its interaction with the Notch downstream effector CBL/CSF1 [42] and to play a crucial role in triggering the switch from KSHV latency to lytic cycle through interfering with the BCL6-vIRF4 axis [43]. In addition to its interaction with USP7, vIRF3 has been shown to promote the degradation of Promyelocytic leukemia nuclear bodies (PML NBs)/nuclear domain 10 (ND10) [44, 45]. PML NBs are known to restrict the replication of several virus families, which have, in turn, evolved mechanisms to counteract this PML-mediated antiviral restriction. Herpesviruses like HCMV, Varicella-Zoster virus (VZV), Epstein-Barr virus (EBV) and HSV1 counteract the antiviral function of PML NB by either interfering with PML NB-associated proteins like Daxx and Sp100, or with PML SUMOylation, leading to the disruption of these cellular antiviral structures [46–56]. For KSHV, it was shown that vIRF3, RTA and ORF75 are antagonists of PML NBs leading to their SUMO-dependent and proteasome-mediated degradation or to the disappearance or the dispersion of PML NB-associated proteins like ATRX, Sp100 and Daxx [44, 45, 57, 58].

The present study focuses on the second vIRF protein, vIRF2, which has previously been shown to interact directly or indirectly with IRF1/2/3/8/9, p65, p300, PKR, Caspase-3 as well as with STAT1, and which could therefore interfere with the cellular IFN pathway, apoptosis, activation-induced cell death (AICD) and the PI3K/Akt signaling pathway [35, 39, 59–63]. In addition to the full-length vIRF2 protein translated from a spliced mRNA including the two exons K11.1 and K11, the existence of a smaller protein generated only from the unspliced K11.1/K11 mRNA and therefore the K11.1 exon has been reported but is controversial [26, 64, 65]. Expression of the spliced vIRF2 mRNA has previously been reported to occur only in the lytic replication cycle in different KSHV positive cell lines [26, 65–68]. Recently, it was shown that vIRF2 is able to bind the promoter regions of different cellular genes by means of a DNA-binding domain (DBD; aa 7–114); a nuclear localization signal (aa146-159) has been identified at the N-terminal end of the vIRF2 protein [36, 69].

Most previous studies on the vIRF2 protein have examined its function by transfecting an expression vector for the spliced vIRF2 mRNA. In this study we investigated the function of vIRF2 during KSHV lytic and latent replication. We show that the vIRF2 protein dampens KSHV early viral gene expression in endothelial cells. Our results suggest that this regulation of the viral life cycle might be connected to the vIRF2-mediated activation of IFN-induced cellular proteins that restrict KSHV lytic gene expression.

## Results

### KSHV infection promotes PML NB formation and PML restricts KSHV lytic reactivation

**Fig 1:**
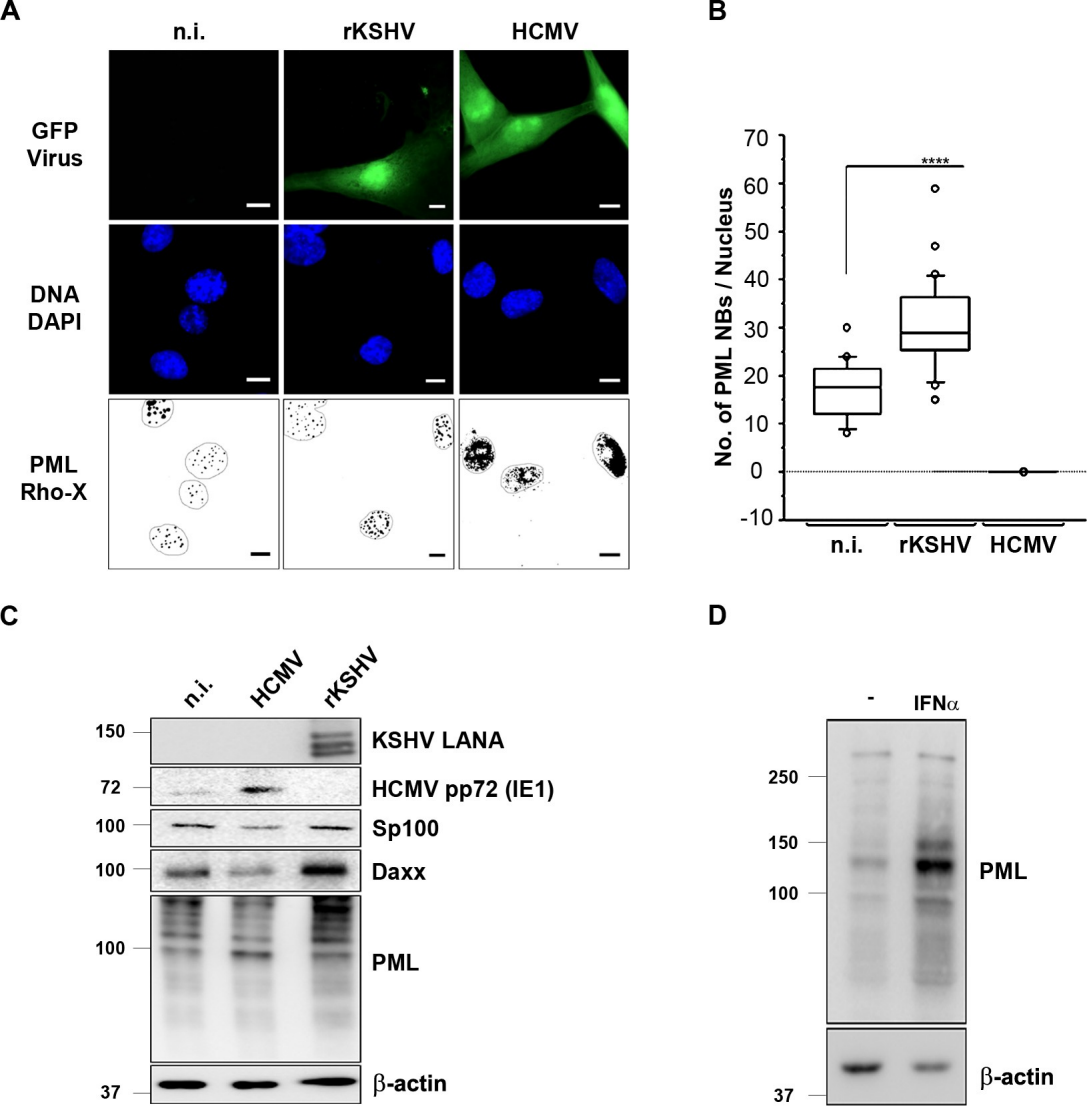
KSHV infection increases the number of PML nuclear bodies and associated proteins in endothelial cells. (**A**) HUVECs were either infected with rKSHV.219 at an MOI of 20, or HCMV at an MOI 3, or left uninfected (n.i.). 48 h after infection cells were fixed and processed for IFA staining. Infected cells express GFP, PML was detected with a mouse anti-PML antibody and a goat anti-mouse IgG Lissamine Rhodamine (LRSC)-conjugated secondary antibody and DNA was stained with DAPI. Images were acquired using a Carl Zeiss microscope at 100x magnification. For better visualization PML NBs are shown as black dots on a white background and the nuclei were encircled. Bars, 10 μm. (**B**) For the quantification of PML NBs upon KSHV and HCMV infection, PML NBs were counted in at least 50 cells per sample and a Mann Whitney U test was performed. Boxes indicate 25th to 75th percentile; the central line inside each box indicates the median and the whisker illustrate the 5th and 95th percentile. The dots indicate the outliers. (**C**) Protein expression of infection markers and PML NBs-associated proteins was analyzed by WB 48 h post infection of HUVECs infected with either KSHV or HCMV. (**D**) HEK-293 cells were treated with IFN-α for 72 h and PML protein expression was analyzed by WB.

When infecting primary human umbilical vein endothelial cells (HUVEC) with KSHV we noted that, in contrast to other herpesviruses such as HCMV, which is known to degrade PML NBs [46–48, 53, 55, 56], and the previously reported ability of KSHV vIRF3 to do the same [44, 45], KSHV does not disrupt these nuclear structures (Fig 1A). While HCMV infection leads to a diffuse distribution of PML staining, infection of HUVECs with KSHV significantly increased the number of discrete PML NBs in infected cells in comparison to uninfected cells (Fig 1A and 1B). In accordance with this immunofluorescence experiment, protein levels, as measured on western blots, of PML itself and of the PML NB components Sp100 and Daxx, decreased after HCMV infection, but increased after KSHV infection of primary endothelial cells (Fig 1C). This KSHV-induced increase in PML and Daxx protein levels and in the number of PML NBs is reminiscent of the effect of IFN treatment, which is shown in Fig 1D and has been described in many previous studies [70–72].

### The KSHV vIRF2 protein increases PML expression

**Fig 2:**
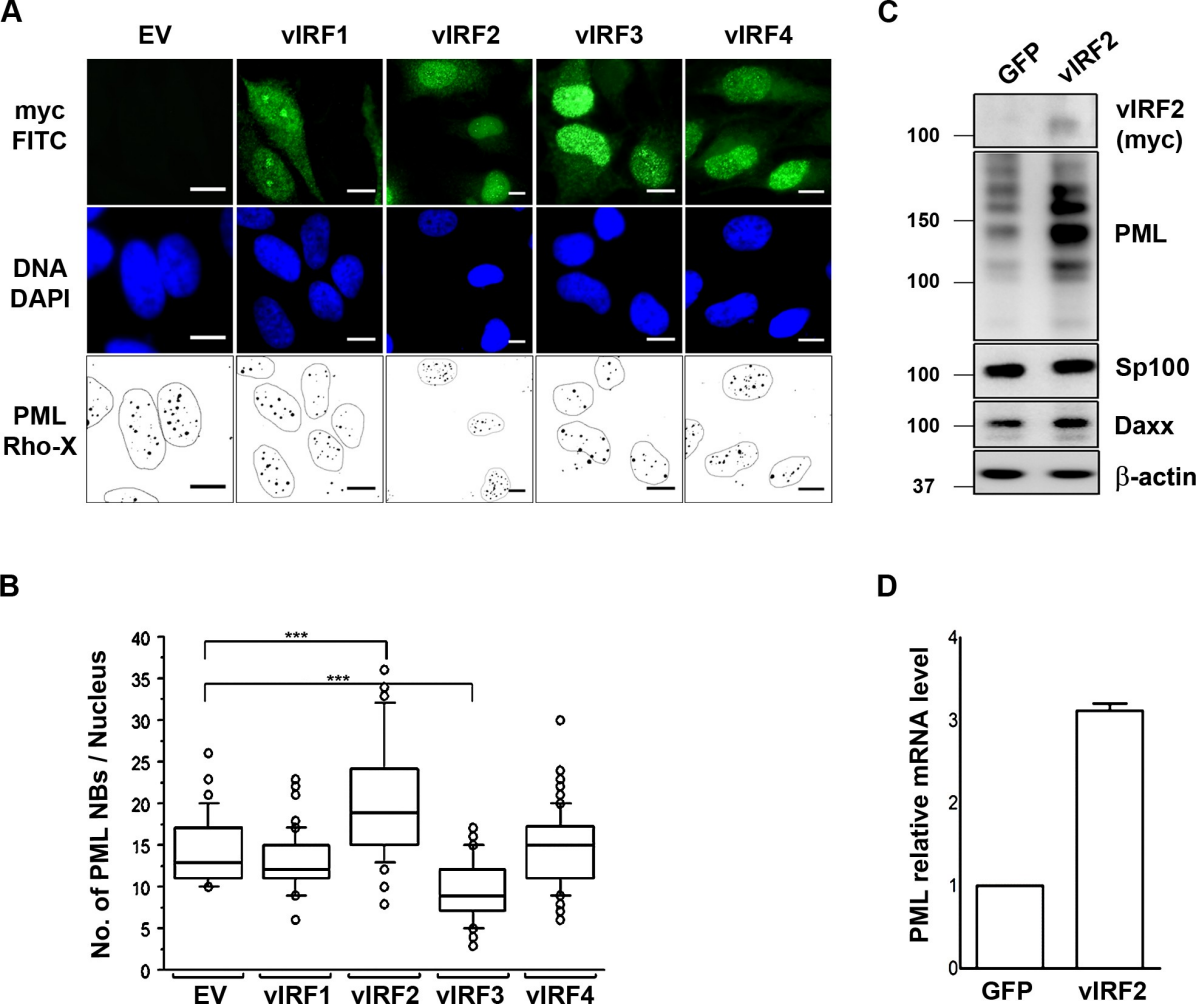
Effects of KSHV vIRF proteins on PML NBs. **(A**) HeLa Cells were transfected with 1 μg of either the control vector or one of the vIRF expressing constructs and were fixed 36 h after transfection for IFA. Transfected cells express GFP, PML was detected with a mouse anti-PML antibody and a goat anti-mouse IgG Lissamine Rhodamine (LRSC)-conjugated secondary antibody and DNA was stained with DAPI. Images were acquired using a Carl Zeiss microscope at 100x magnification. For better visualization PML NBs are shown as black dots on a white background and the nuclei were encircled. Bars, 10 μm. **(B)** Quantification of immunofluorescence data from A, PML NBs were counted in at least 100 cells per each construct and a Man Whitney U test was performed to determine significance. Boxes indicate 25th to 75th percentile; central line inside each box indicates the median and the whisker illustrates the 5th and 95th percentile. The dots indicate the outliers. **(C)** HUVECs were transduced with either the control or the vIRF2 expressing lentiviral vector and 36 h after transduction cells were lysed and protein expression was analyzed by WB. **(D)** HUVECs were transduced with either the control or vIRF2 expressing lentivirus and 36 h later cells were lysed for RNA extraction. PML mRNA was quantified by reverse transcription following qPCR using dually labeled probes (Taqman).

Such an ‘IFN-like’ phenotype would be in keeping with the previously noted upregulation of interferon-stimulated cellular genes following KSHV infection [73–75]. However, several KSHV vIRFs have been reported to act as interferon antagonists that counteract the induction of the IFN pathway or the expression of ISGs [35, 39, 59, 61, 63], vIRF3 has been shown to mediate the degradation of PML NBs [44, 45]. We therefore compared the effect of all four KSHV vIRF proteins on PML NB formation by transfecting expression vectors for tagged cDNAs of all vIRFs into HeLa cells and analyzing the number of PML NBs by immunofluorescence. As described for B cells [44, 45], overexpression of vIRF3 causes a reduction in the number of PML NBs also in HeLa cells (Fig 2A and 2B). In contrast, while vIRF1 and vIRF4 did not affect the number of PML NB in transfected cells, the overexpression of vIRF2 led to an increase in PML NB formation compared to the control vector (Fig 2A and 2B). We then transduced HUVECs with either a control or a vIRF2 expressing lentiviral vector and found PML protein levels as well as mRNA levels to be increased in the presence of vIRF2 (Fig 2C and 2D). These results suggest that, in contrast to vIRF3, vIRF2 increases the expression of PML at the transcriptional level and thereby PML NB numbers in a similar manner as is seen following the infection of primary endothelial cells with KSHV (Fig 1A–1C) or treatment with IFNα in epithelial cells (Fig 1D).

### Expression of vIRF2 in KSHV-infected cells

**Fig 3:**
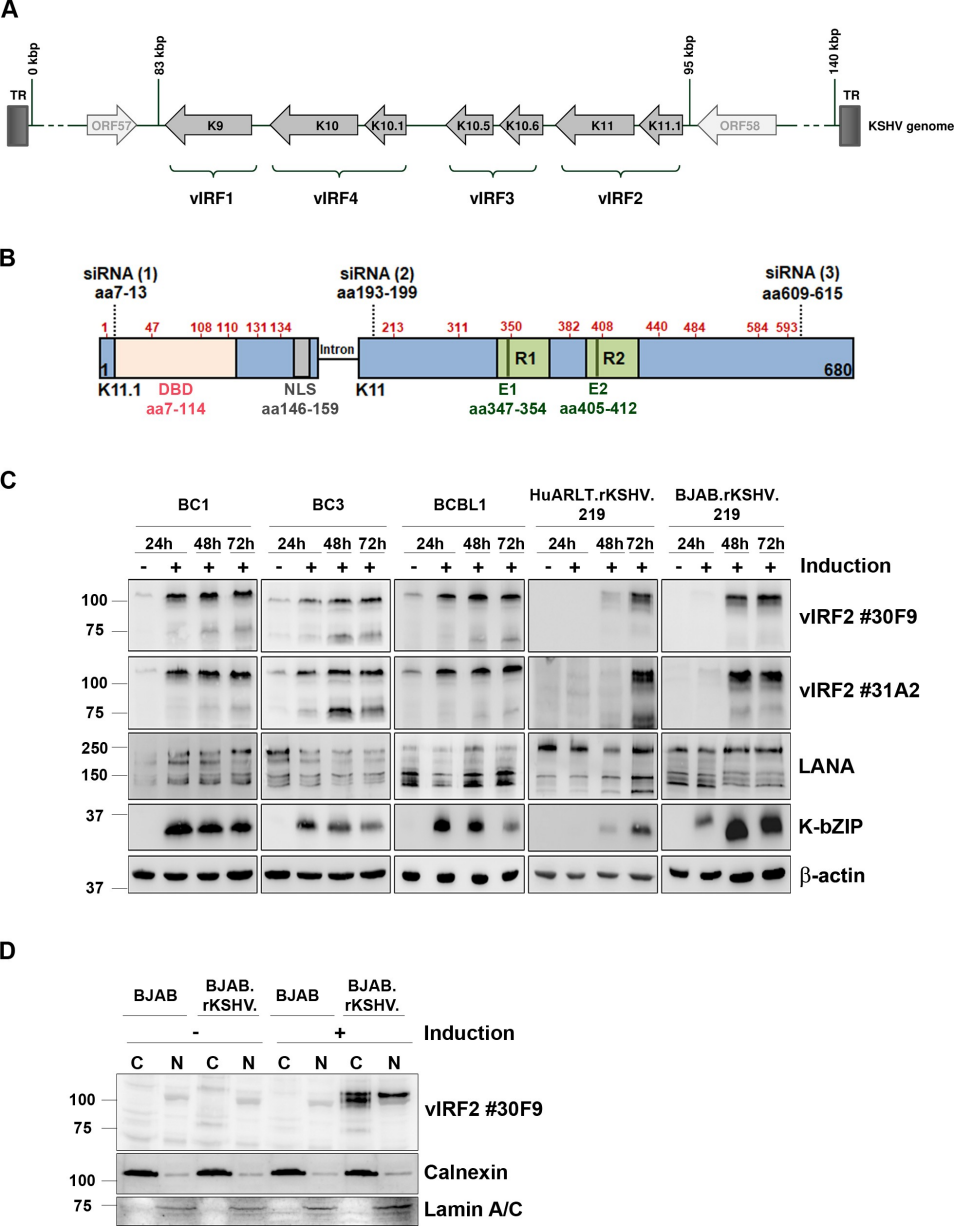
Expression of vIRF2 in KSHV-infected cells. (**A**) The vIRF genes are located between ORF57 and ORF58 in the KSHV genome. The vIRF2 protein is encoded by the two exons K11.1 and K11. (**B**) Schematic diagram of the vIRF2 open reading frame, shown in the opposite orientation to its position in the viral genome. ATG start codons are indicated by red amino acid numbers at the positions aa 1, 47, 103, 108 110, 131, 134 in K11.1 and aa 213, 311, 350, 382, 408, 440, 484, 584, 593 in K11. Dotted lines indicate binding sites for siRNAs used in this study: aa7-13 (1), aa193-199 (2) and aa609-615 (3), DBD: DNA binding domain (aa7-114), NLS: nuclear localization signal (aa146-159), R1/R2: repeat region 1/2, E1/2: antibody epitopes. (**C**) Expression of vIRF2 in different KSHV positive cell lines. The lytic cycle in the PEL-derived cell lines BC1, BC3 and BCBL1 was induced by 100 ng/ml TPA, HuARLT.rKSHV.219 cells were induced using 1.67 mM SB and 10% tissue culture supernatant containing RTA-expressing baculovirus, the BJAB.rKSHV.219 using 2.5 μg/ml goat anti human-IgM. At the indicated time points after induction the cells were lysed and protein expression was analyzed by WB. (**D**) Subcellular localization of vIRF2 was analyzed by nuclear and cytoplasmic extraction of BJAB or BJAB.rKSHV.219 cells with or without induction of the lytic cycle using 2.5 μg/ml goat anti human-IgM. WB analysis was performed for vIRF2, Calnexin (cytoplasmic marker; C) and Lamin A/C (nuclear marker; N).

This observation would seem to be at odds with the previously reported ability of transfected vIRF2 to antagonize the activation of the IFNβ promoter and the type I interferon-induced JAK-STAT signal transduction cascade [61, 63]. We therefore wanted to investigate the role of vIRF2 in KSHV-infected cells. To follow the expression of vIRF2 in KSHV-infected cells we generated a monoclonal antibody against a recombinant vIRF2 fragment corresponding to the entire K11 exon. We expressed the K11 exon as a GST-K11-6xHis fusion protein in *E*. *coli* and immunized mice to obtain two hybridoma cell lines producing IgG2b/κ monoclonal antibodies #30F9 and #31A2. We mapped the epitopes recognized by these two monoclonal antibodies using a peptide array that spanned the K11 protein sequence (S1 Fig). Both monoclonal antibodies recognized the same repeated epitope (QGPMQSEG) located at position aa347-354 and aa405-412 within the repeated sequence ELLCETASPQGPMQSEGGEEGSTES in the regions R1 and R2 of vIRF2, as illustrated in Fig 3B and S1 Fig. In addition, we observed a more weakly reactive third epitope (S1 Fig, peptides 41–44) within the vIRF2 protein, whose sequence is only partially homologous to that of the first two epitopes and which might be detected by our two monoclonal antibodies which a lower affinity.

Using the two monoclonal antibodies we were able to detect vIRF2 in KSHV-infected PEL cell lines as well as in *in vitro* infected endothelial and B cells (Fig 3C). The vIRF2 expression pattern in the PEL-derived cell lines BC1, BC3 and BCBL1 was similar, with a low basal expression that increased over time following induction of the lytic replication cycle. We also used HuARLT.rKSHV.219 cells, a conditionally immortalized human endothelial cell line derived from HUVECs expressing the doxycycline-inducible simian virus 40 (SV40) large T antigen (TAg) and a human telomerase reverse transcriptase (hTert), as well as the B cell line BJAB.rKSHV.219, which are both latently infected with recombinant KSHV. Both cell lines showed vIRF2 expression only after lytic induction. The low basal vIRF2 expression in the three PEL cell lines (Fig 3C) may therefore reflect that a small percentage of these cells shows lytic gene expression even in the absence of external stimuli. Interestingly, we could detect several different bands for the vIRF2 protein, of which the full-length form with the highest molecular weight (~ 110 kDa) showed the strongest intensity. We also investigated the subcellular localization of vIRF2 by a fractionation assay on KSHV positive BJAB.rKSHV.219 cells after lytic cycle induction (Fig 3D). While two adjacent bands of the molecular weight expected for the full-length vIRF2 were detected in the cytoplasm, the nucleus contained mainly the upper of the two bands.

These observations confirm that KSHV vIRF2 is expressed at a low basal level in latent PEL cell lines and strongly expressed early after activation of the lytic replication cycle, as predicted from previous studies of its mRNA [26]. Our observation that vIRF2 is found both in the nucleus and cytoplasm of KSHV-infected cells, is in keeping with overexpression studies that used an expression vector for the spliced K11.1/K11 cDNA [69].

### KSHV vIRF2 inhibits early lytic protein expression in endothelial cells

**Fig 4:**
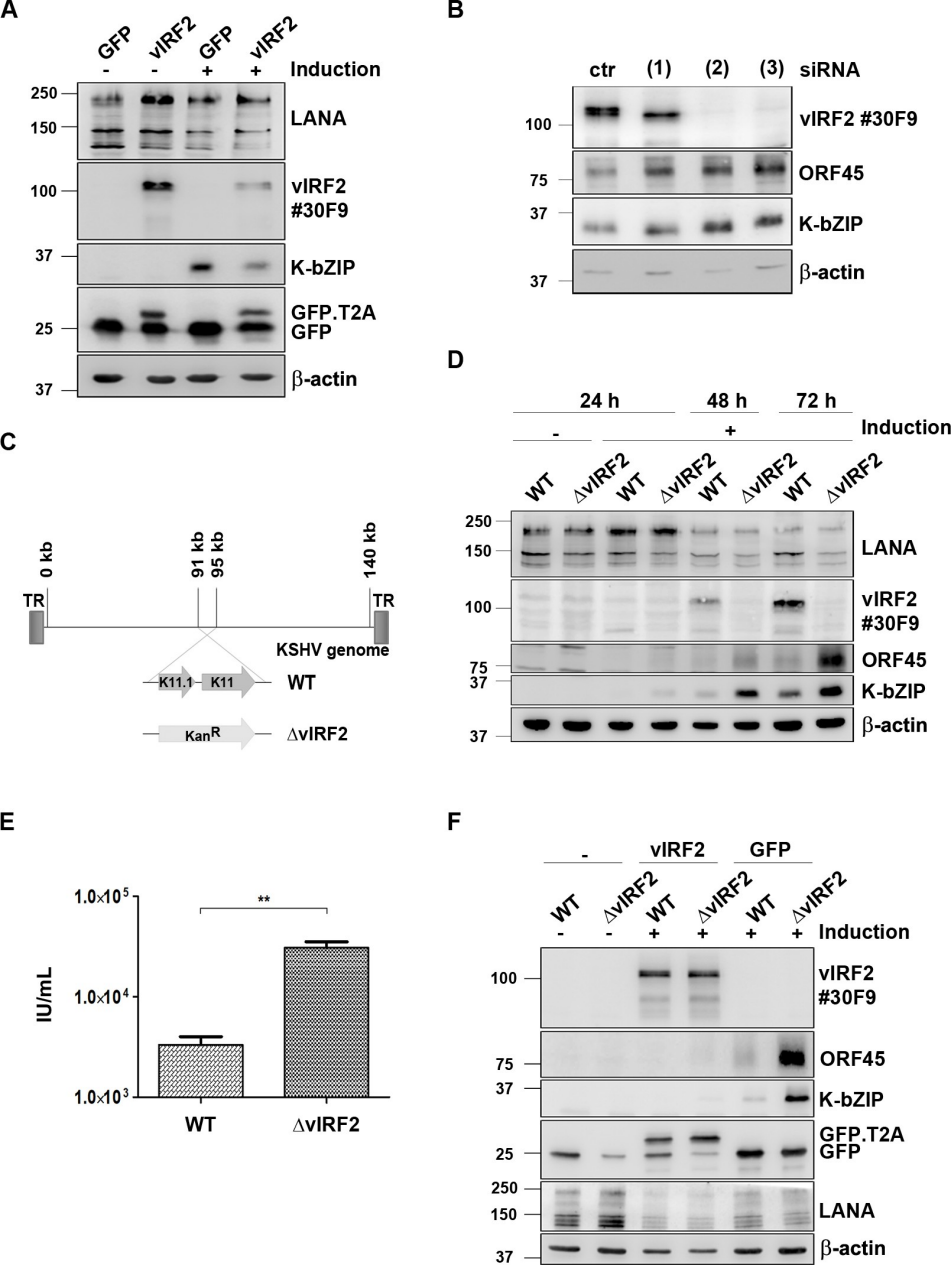
KSHV vIRF2 suppresses KSHV lytic protein expression upon reactivation. (**A**) HuARLT.rKSHV.219 cells were transduced with either the control GFP or the GFP-T2A-vIRF2 encoding lentivirus. 24 h later, the lytic cycle was induced by 12.5% tissue culture supernatant containing RTA-expressing baculovirus and 1.25 mM SB. After 48 h cells were lysed and protein expression was analyzed by WB. (**B**) HuARLT.rKSHV.219 cells were microporated with either a non-targeting siRNA (ctr) or three different vIRF2 siRNAs and 24 h later the lytic cycle was induced with 10% tissue culture supernatant containing RTA-expressing baculovirus and 1.67 mM SB. The cells were lysed 72 h after induction and protein expression was analyzed by WB. (**C**) Schematic diagram of the KSHV genome in the BAC16 vector, comparison of the BAC16.KSHV.WT and the BAC16.KSHV.ΔvIRF2 genome in which the whole vIRF2 gene was replaced with a kanamycin resistance gene (Kan^R^). (**D**) Stable HuARLT.BAC16.KSHV.WT and ΔvIRF2 cells were induced using 12.5% tissue culture supernatant containing RTA-expressing baculovirus and 1.67 mM SB. Protein expression was analyzed by WB after lysis of the cells at the indicated time points after induction. (**E**) Supernatants of the reactivated HuARLT.BAC16.KSHV.WT and ΔvIRF2 cells after 72 h were used to determine infectious viral titers on HEK.293 cells. In three independent experiments titers for KSHV.WT-infected cells ranged from 2.3–4.6×10^3^/ml and for KSHV.ΔvIRF2-infected cells from 2.6–4×10^4^/ml. (**F**) HuARLT.BAC16.KSHV.WT and ΔvIRF2 cells were transduced with either the control GFP or the GFP-T2A-vIRF2 encoding lentivirus and the lytic cycle was induced as described in panel A. After 48 h cells were lysed and protein expression was analyzed by WB, using antibodies for the indicated proteins.

Having shown that the vIRF2 protein is expressed during lytic viral replication and that it increases the expression of PML and the number of PML NBs, we wanted to investigate its function in KSHV infected cells. As a first step we overexpressed vIRF2 in the KSHV-infected immortalized endothelial cell line HuARLT.rKSHV.219 [73] via lentiviral transduction and induced the lytic cycle. We could observe a strong inhibition of KSHV early lytic protein expression upon vIRF2 overexpression, as judged by the reduced expression of the KSHV early lytic protein K-bZIP (Fig 4A). In addition, we silenced vIRF2 in the same cell system using three siRNAs targeting different regions of the vIRF2 mRNA, as illustrated in Fig 3B. Using the siRNAs (2) and (3), which target regions within the vIRF2 K11 sequence, the vIRF2 protein expression was suppressed to undetectable levels (Fig 4B, top panel). In contrast, using siRNA (1), which targets vIRF2 mRNA at the 5’ end of exon K11.1, resulted only in the silencing of the protein form with the highest molecular weight (Fig 4B, top panel). We hypothesize that siRNA (1) interferes with the translation of the vIRF2 mRNA from the first translational start codon. Interestingly, the knockdown with any of these three siRNAs led to an increased early lytic gene expression, as shown here for the early lytic proteins K-bZIP and ORF45 (Fig 4B).

To verify a possible role of vIRF2 in lytic cycle regulation with another experimental approach, we used the BAC16 vector [76] to generate a vIRF2 deletion mutant (KSHV.ΔvIRF2) by replacing the ORFs K11 and K11.1 with a kanamycin resistance cassette (Fig 4C). We sequenced the entire BAC16 KSHV.ΔvIRF2 to verify the correct insertion of the kanamycin cassette and the integrity of the rest of the KSHV genome. We then established stable HEK-293.BAC16.KSHV.WT and HEK-293.BAC16.KSHV.ΔvIRF2 cell lines by transfecting, respectively, the BAC16.KSHV.WT and BAC16.KSHV.ΔvIRF2 genomes into HEK-293 cells and selecting stable hygromycin B resistant bulk populations. Following induction of the lytic replication cycle, expression of the vIRF2 protein was detected in the HEK-293.BAC16.KSHV.WT cells but not in the HEK-293.BAC16.KSHV.ΔvIRF2 cell line. There was no detectable difference in the expression level of early lytic proteins upon reactivation in the KSHV.ΔvIRF2-infected cells compared to the KSHV.WT-infected cells (S2 Fig). To investigate the function of vIRF2 in the context of the whole viral genome in endothelial cells, we established stable HuARLT.BAC16.KSHV.WT and HuARLT.BAC16.KSHV.ΔvIRF2 cells by infecting the immortalized endothelial cell line HuARLT with tissue culture supernatants of induced HEK-293.BAC16.KSHV.WT and HEK-293.BAC16.KSHV.ΔvIRF2 cells and selecting hygromycin B resistant bulk populations. Following induction of the lytic cycle, vIRF2 protein expression could be detected only in the HuARLT.BAC16.KSHV.WT cells, but not in the HuARLT.BAC16.KSHV.ΔvIRF2 cells (Fig 4D). In this endothelial cell lines, we observed an increased expression of the lytic proteins K-bZIP and ORF45 in KSHV.ΔvIRF2- compared to KSHV.WT-infected cells following activation of the lytic cycle (Fig 4D). In addition, the viral titer released from induced KSHV.ΔvIRF2-infected HuARLT cells was about 10 fold higher than from induced KSHV.WT-infected cells (Fig 4E). To confirm that the increased lytic gene expression in KSHV.ΔvIRF2-infected HuARLT cells was due to the absence of the vIRF2 protein we complemented vIRF2 by lentiviral transduction in stable HuAR2T.BAC16.KSHV.WT and HuAR2T.BAC16.KSHV.ΔvIRF2 cells and induced the lytic replication cycle. The vIRF2 overexpression led to an inhibition of early viral protein expression in the HuAR2T.BAC16.WT and also in the HuAR2T.BAC16.ΔvIRF2 cells (Fig 4F). This experiment showed that overexpressed vIRF2 is able to suppress early viral protein expression not only in KSHV.WT-infected endothelial cells (as already shown in Fig 4A), but also the increased lytic gene expression seen in KSHV.ΔvIRF2-infected endothelial cells.

Together, these results show that, in endothelial cells, the deletion of vIRF2 from the viral genome or its suppression by siRNA promotes early viral protein expression and, in the case of the vIRF2 deletion, the production of viral progeny.

### The KSHV vIRF2 protein curtails lytic protein expression in newly infected endothelial cells

After infection of the host cell, KSHV initially expresses lytic viral proteins but then switches off their expression as latency is established. Latency represents an important step during the KSHV life cycle and is indispensable for KSHV pathogenesis.

**Fig 5:**
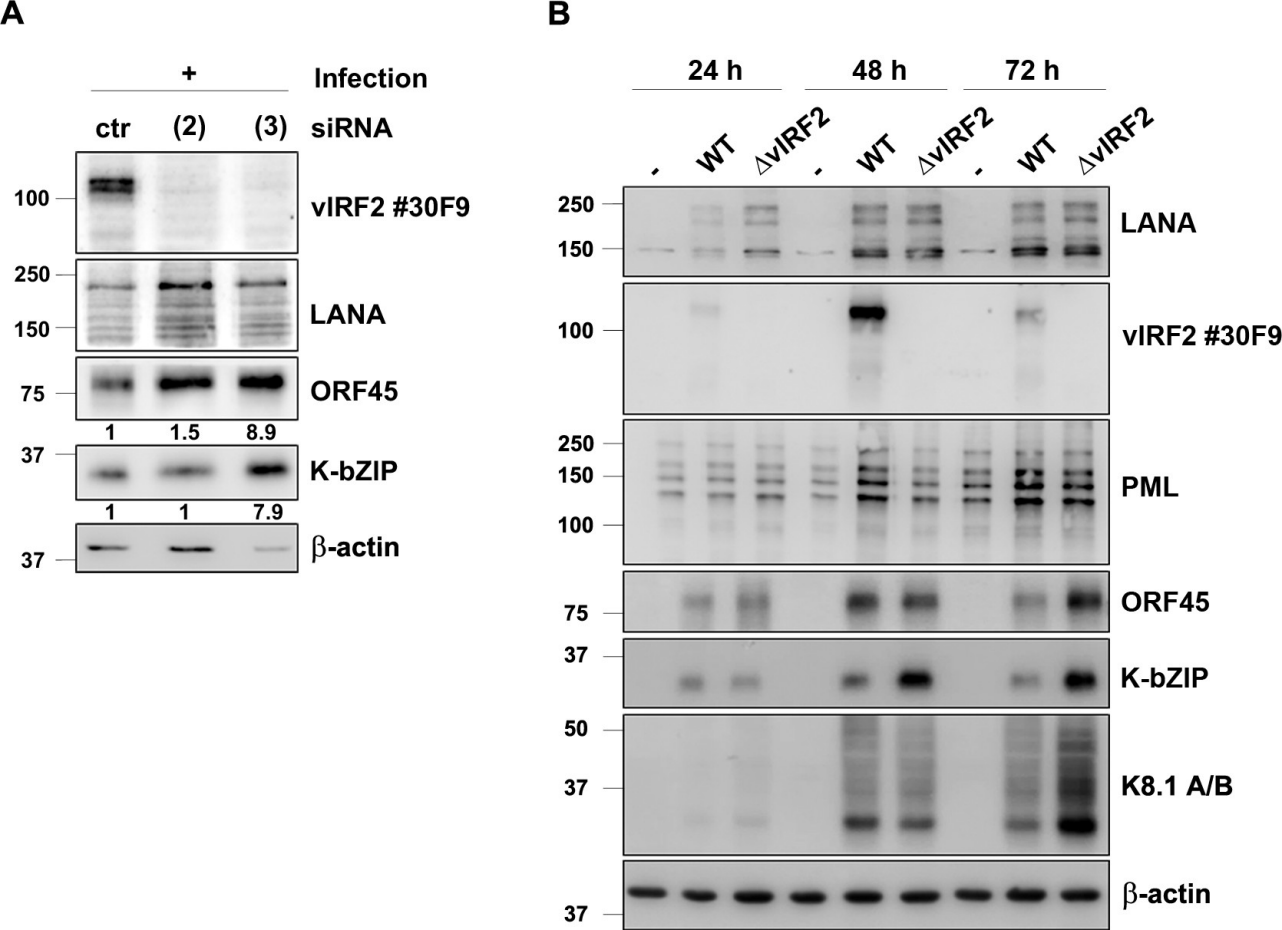
KSHV vIRF2 inhibits KSHV lytic protein expression after *de novo* infection. (**A**) HuARLT cells were microporated with either a non-targeting siRNA (ctr) or with two different vIRF2 siRNAs (see Figs 3 and 4) and 24 h later cells were infected with rKSHV at a MOI of 5 (titer determined on HEK-293 cells). The cells were lysed 72 h after induction and protein expression was analyzed by WB. ORF45 and K-bZIP protein levels were quantified and normalized to β-actin levels by using the Image studio software. Relative expression levels (in comparison to those seen with the control siRNA) are indicated below the respective blots. (**B**) HuARLT cells were infected with either the KSHV.WT virus or the KSHV.ΔvIRF2 virus at a MOI of 5. The cells were lysed at the indicated time points and protein expression was analyzed by WB.

In view of our observations in experiments on lytic reactivation from latency, we were curious if the vIRF2 protein is also involved in the regulation of lytic viral protein expression following a new infection. For this, we infected HuARLT cells that had been microporated with two siRNAs targeting ORF K11 (see above, Fig 3B) with a recombinant KSHV. We observed an increase in the expression of the lytic proteins K-bZIP and ORF45 upon the transient knockdown of vIRF2 by siRNA (3) which targets vIRF2 mRNA at the C-terminal end (Fig 5A and Fig 3B). Using another siRNA, directed against a region in the center of the vIRF2 mRNA, we could only observe an increase in ORF45, but not of K-bZIP expression in comparison to the control siRNA (Fig 5A).

To confirm this result, we used cell free KSHV.WT or KSHV.ΔvIRF2 virus, which was generated in reactivated HEK-293.BAC16.KSHV.WT and HEK-293.BAC16.KSHV.ΔvIRF2 cell lines (see S2 Fig), to infect HuARLT cells. We verified equal infection levels in HuARLT cells by western blot for the latent KSHV LANA protein (Fig 5B, top panel). KSHV.WT-infected cells showed the strongest expression of the early lytic KSHV proteins K-bZIP and ORF45 48 hours after infection, which was followed by a decline at 72 hours, indicating the onset of latency establishment. In contrast, cells which were infected with the KSHV.ΔvIRF2 virus showed an increased and prolonged early lytic protein expression which did not decline at 72 hours after infection. Similarly, the expression of the late lytic KSHV glycoprotein K8.1 (Fig 5B, K8.1 A/B panel) was increased in KSHV.ΔvIRF2- compared to KSHV.WT-infected cells at 72 hours after infection. In addition, we observed again that PML protein expression is strongly increased upon infection with the KSHV.WT virus but much less after infection with the vIRF2 knockout virus (Fig 5B, PML panel).

These data indicate that vIRF2 not only inhibits KSHV lytic gene expression during reactivation but also after *de novo* infection.

### Domains of vIRF2 required for the restriction of KSHV early lytic protein expression in endothelial cells

As mentioned above, two different protein forms of vIRF2 have previously been described. Among them is the short variant encoded by the K11.1 exon only and a protein of approximately 110 kDa apparent molecular weight resulting from the translation of the spliced K11.1/ K11 mRNA [26, 35, 59–61, 63, 65, 77]. Interestingly, by using our newly produced monoclonal antibodies against vIRF2, we were able to detect four additional protein forms in different KSHV positive cell lines (Fig 3C).

**Fig 6:**
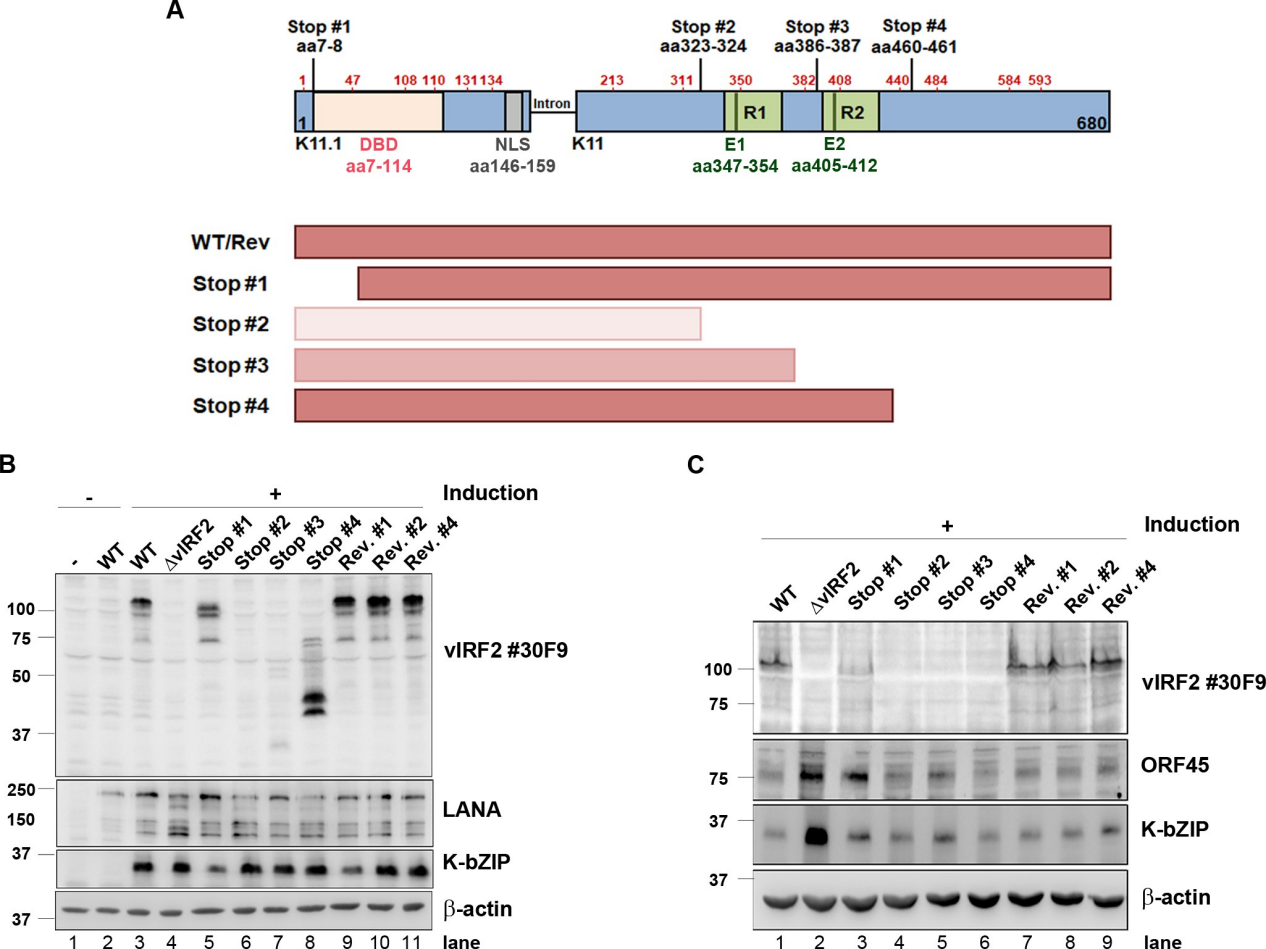
Domains of vIRF2 required for the inhibition of early lytic KSHV protein expression. (**A**) Schematic diagram of the possible vIRF2 protein forms expressed from KSHV ORFs K11.1 and K11 in different vIRF2 double stop mutants compared to the WT-infected cells. Methionine residues are indicated by red amino acid numbers at the positions aa1, 47, 103, 110, 131, 134 in K11.1 and aa213, 311, 350, 382, 408, 440, 484, 584, 593 in K11. Lines indicate double stop mutations at the positions aa7-8, aa323-324, aa386-387 and aa460-461, DBD: DNA binding domain (aa7-114), NLS: nuclear localization signal (aa146-159), R1/R2: repeat region 1/2, E1/E2: antibody epitope. (**B**) The different stable HEK-293.BAC16 cell lines were induced using 10% tissue culture supernatant containing RTA-expressing baculovirus and 1.67 mM SB. Protein expression was analyzed 72 h post induction by WB after lysis. (**C**) The different stable HuARLT.BAC16 cells were induced using 12.5% tissue culture supernatant containing RTA-expressing baculovirus and 1.67 mM SB for 72 h. Protein expression was analyzed by WB after lysis of the cells. Stop #1, aa7-8; Stop #2, aa323-324; Stop #3, aa386-387; Stop #4, aa460-461. Rev. #1, revertant to Stop #1; Rev. #2, revertant to Stop #2; Rev. #4, revertant to Stop #4.

To further understand which forms and parts of the vIRF2 protein are necessary and/or sufficient for its regulatory function during KSHV replication, we generated four different KSHV mutants (Stop#1—Stop#4) by introducing double stop codons at different positions within the vIRF2 sequence in the BAC16 backbone using *En passant* mutagenesis (Fig 6A). We also constructed the corresponding revertants (Rev#1, 2 and 4) with the exception of revertant #3, which failed repeatedly for technical reasons. We then generated stable HEK-293.BAC16.KSHV cell lines with these mutants and their revertants, as mentioned before. Following reactivation, we found that all three revertant cell lines express the same vIRF2 pattern as KSHV.WT-infected cells (Fig 6B, lane 9–11, 3). As already observed before, there was no expression in the KSHV.ΔvIRF2 cell line (Fig 6B, lane 4). In addition no vIRF2 protein was detected in the case of the KSHV.vIRF2.Stop#2 cell line (Fig 6B, lane 6), in keeping with the position of the antibody epitopes E1/E2 (S1 Fig) downstream of the inserted stop codons (Fig 6A). It is, however, likely that KSHV.vIRF2.Stop#2 expresses a C-terminally truncated vIRF2 (aa1-322) as illustrated in Fig 6A. In contrast, the KSHV.vIRF2.Stop mutants #1, #3 and #4 showed several bands of different molecular weight that were detected by the vIRF2 antibody (Fig 6B, lane 5, 7, 8). We assume that these bands result from translational initiation at internal methionine codons, indicated by red numbers in the diagram in Fig 6A. Thus, the protein bands with the highest molecular weight observed in the case of the KSHV.vIRF2.Stop#1 and KSHV.vIRF2.Stop#4 mutants could initiate at a methionine in position aa47 of the vIRF2 sequence, and bands of lower apparent molecular weight could be translated from other internal methionine codons. The weak immunoreactive band at ~ 30 kDa, detected with the monoclonal antibody in the case of KSHV.vIRF2.Stop#3, suggests that either this antibody mainly detects epitope E2 in the second repeat in the context of the entire vIRF2 protein, in spite of reacting with epitopes E1 and E2 on the peptide array (S1 Fig) or the vIRF2 protein expressed by the Stop#3 mutant could be unstable. Neither the HEK-293.KSHV.ΔvIRF2 cell line, nor the HEK-293.KSHV.vIRF2.Stop#2-#4 cell lines differed significantly from the HEK-293.KSHV.WT cell line with regard to the expression of the KSHV lytic protein K-bZIP (Fig 6B). We interpret the lower levels of K-bZIP expression observed with Stop mutant #1 as not being due to the absence of the full-length vIRF2 form, as it is also seen in the corresponding revertant #1 (Fig 6B, lanes 5,9).

To investigate if the individual KSHV.vIRF2 stop mutants differed in their reactivation potential in endothelial cells, we established stable HuARLT populations infected with KSHV.WT or the KSHV.vIRF2 stop mutants and the corresponding revertants. Interestingly, while the complete deletion of vIRF2 led to an increased expression of KSHV K-bZIP and ORF45 as noted before (Fig 4C, Fig 6C lane 2), KSHV.vIRF2 stop mutants #2–4 did not increase the levels of these lytic proteins, while Stop mutant #1 showed an increase in ORF45 but not K-bZIP expression in endothelial cells (Fig 6C). This indicates that the truncated vIRF2 protein forms expressed by stop mutants #2-#4 were still functional and sufficient to maintain the WT phenotype. In particular, these results suggest that the first 460 amino acids of the vIRF2 protein, which are likely produced by KSHV.vIRF2.Stop#4, and probably the first 322 amino acids, which are likely produced by KSHV.vIRF2.Stop#2 but are not detected by our antibody, are sufficient to restrict KSHV lytic gene expression. The phenotype observed with the Stop# 1 (increase in ORF45 but not K-bZIP expression) is more difficult to interpret but could suggest that the first 47 amino acids, which are likely lacking in KSHV.vIRF2.Stop#1, are required for vIRF2 to restrict lytic gene expression.

### The KSHV vIRF2 protein is a transcriptional regulator of interferon-stimulated cellular genes

Having shown that vIRF2 increases the expression of cellular antiviral factors like PML (Fig 2) and that it has an inhibitory effect on KSHV lytic replication (Fig 4 and Fig 5) we wondered if vIRF2 regulates the transcription of cellular genes.

To address this question we performed a microarray-based mRNA expression analysis. We used empty HuARLT cells, the HuARLT.BAC16.KSHV.WT- and the HuARLT.BAC16.KSHV.ΔvIRF2-infected cells, treated them with the reactivation cocktail for 48 h and extracted mRNA, which was then analyzed using a gene expression microarray. The original microarray data were filtered for up- or downregulated genes that showed a 2 fold or higher difference between KSHV.ΔvIRF2-infected and KSHV.WT-infected cells following lytic reactivation in two independent experiments. This yielded 434 cellular genes that could be regulated, directly or indirectly, by vIRF2 in KSHV-infected cells. This list was sorted for their involvement in biological processes using Gene Ontology. This yielded 51 genes known to be involved in innate or intrinsic defense response (Table 1). Among these vIRF2-regulated genes were the three IFN-induced proteins with tetratricopeptide repeats (IFIT1, 2 and 3), whose expression was downregulated in the KSHV.ΔvIRF2-infected compared to KSHV.WT-infected cells. The average fold differences ranged from 3.1 for IFIT1, 3.8 for IFIT3 and 6.1 for IFIT2.

**Table 1:** List of vIRF2-dependent cellular genes involved in defense response.

Gene Symbol	Gene Description	Fold Change	
		Exp. #1	Exp. #2
FGB	Fibrinogen beta chain	32.0	57.5
CLEC1B	C-type lectin domain family 1 member B	31.1	30.8
FGA	Fibrinogen alpha chain	3.2	10.7
UBD	Ubiquitin D	8.5	4.3
CLU	Clusterin	3.9	5.0
CXCR4	Chemokine (C-X-C motif) receptor 4	3.3	5.5
LYPD1	LY6/PLAUR domain containing 1	3.0	5.7
CD36	CD36 molecule (thrombospondin receptor)	3.9	3.6
DEFB1	Defensin beta 1	4.1	3.1
CLEC1A	C-type lectin domain family 1 member A	2.2	4.7
CCL15	Chemokine (C-C motif) ligand 15	3.9	3.2
KIT	v-kit Hardy-Zuckerman 4 feline sarcoma viral oncogene homolog	2.4	4.0
PTGS1	Prostaglandin-endoperoxide synthase 1	2.8	3.6
LY75	Lymphocyte antigen 75	2.8	3.3
KALRN	Kalirin	2.3	3.8
TREM1	Triggering receptor expressed on myeloid cells 1	3.2	2.7
IL1A	Interleukin 1 alpha	2.5	2.3
IL1B	Interleukin 1 beta	2.1	2.2
HIST2H2BE	Histone H2B type 2-E	2.3	2.2
SERPING1	Serpin peptidase inhibitor, clade G (C1 inhibitor) member 1	2.0	2.1
DEF6	Differentially expressed in FDCP 6 homolog (mouse)	-2.1	-2.3
TDGF1	Teratocarcinoma-derived growth factor 1	-2.3	-2.1
F3	Coagulation factor III (thromboplastin, tissue factor)	-2.4	-2.3
C4BPB	Complement component 4 binding protein beta	-2.4	-2.1
CFH	Complement factor H	-2.3	-2.3
CXCL8	Chemokine (C-X-C motif) ligand 8	-2.2	-2.4
KCNN4	Potassium intermediate/small conductance calcium-activated channel subfamily N member 4	-2.5	-2.4
BMP6	Bone morphogenetic protein 6	-2.4	-2.7
VGF	VGF nerve growth factor inducible	-2.3	-2.7
ULBP1	UL16 binding protein 1	-2.5	-2.5
NLRP3	NLR family, pyrin domain containing 3 (NLRP3)	-3.0	-2.1
HERC5	HECT and RLD domain containing E3 ubiquitin protein ligase 5	-2.3	-3.0
CCL20	Chemokine (C-C motif) ligand 20	-3.3	-2.4
ADAMTS5	ADAM metallopeptidase with thrombospondin type 1 motif 5	-3.3	-2.5
IFIT1	Interferon-induced protein with tetratricopeptide repeats 1	-2.6	-3.5
NCF2	Neutrophil cytosolic factor 2	-3.9	-2.3
LOXL3	Lysyl oxidase-like 3	-3.7	-2.7
KLRC3	Killer cell lectin-like receptor subfamily C member 3	-3.0	-3.9
CAMP	Cathelicidin antimicrobial peptide	-3.1	-4.1
CXCL6	Chemokine (C-X-C motif) ligand 6	-4.2	-3.1
SLAMF7	SLAM family member 7	-2.9	-4.4
SIGLEC15	Sialic acid binding Ig-like lectin 15	-3.8	-3.5
IFIT3	Interferon-induced protein with tetratricopeptide repeats 3	-2.7	-4.8
IL33	Interleukin 33	-5.5	-2.5
CCR10	Chemokine (C-C motif) receptor 10	-5.3	-2.9
ZC3HAV1	Zinc finger CCCH-type antiviral 1	-3.8	-4.6
GGT5	Gamma-glutamyltransferase 5	-4.7	-3.8
OLR1	Oxidized low density lipoprotein (lectin-like) receptor 1	-5.2	-5.1
IFIT2	Interferon-induced protein with tetratricopeptide repeats 2	-5.7	-6.5
STATH	Statherin	-5.3	-8.8
CGA	Glycoprotein hormones, alpha polypeptide	-9.2	-6.0

Cellular gene expression in KSHV.WT- vs. KSHV.ΔvIRF2-infected HuARLT cells undergoing lytic reactivation was measured by gene expression array. The values shown in the table represent ratios of intensity values (KSHV.ΔvIRF2-infected/KSHV.WT-infected) in lytically induced cells divided by the same ratio (KSHV.ΔvIRF2-infected/KSHV.WT-infected) in uninduced cells in two independent experiments. Negative values indicate genes that are upregulated by vIRF2, positive values those that are downregulated by vIRF2 in KSHV-infected cells.

The vIRF2-regulated ISGs IFIT1, 2 and 3 are known to be involved in the cellular antiviral response. Through their IFN-dependent expression, IFIT proteins are induced upon infection with RNA and DNA viruses and exhibit antiviral functions by interfering with the viral life cycle at different steps. It is known that they antagonize RNA viruses like Vesicular stomatitis virus (VSV), Hepatitis C Virus (HCV), West Nile virus (WNV), Sendai Virus (SeV), Japanese encephalitis Virus (JEV) as well as two dsDNA viruses, the Human Papillomavirus (HPV) and HCMV [78–89].

**Fig 7:**
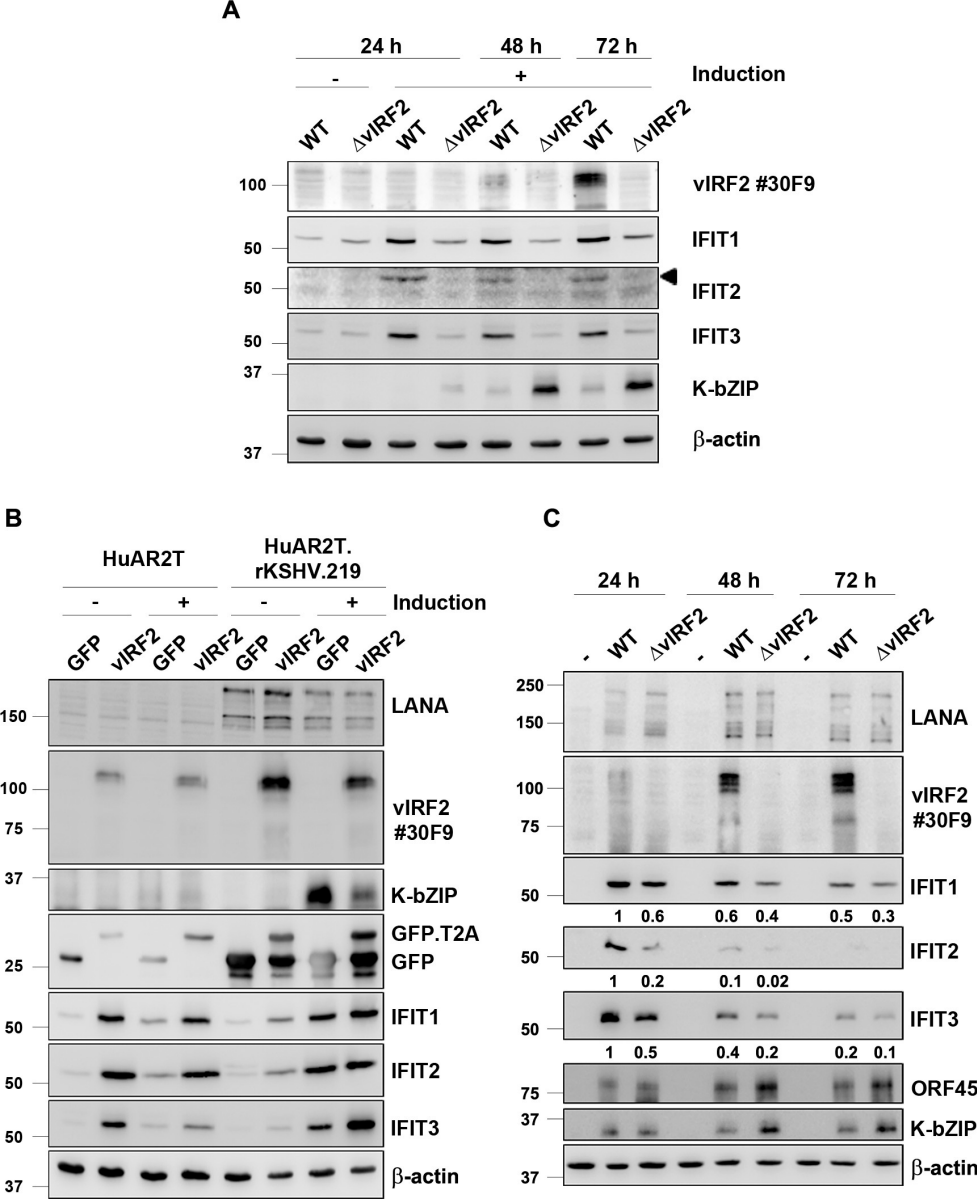
vIRF2 induces IFN-regulated proteins with tetratricopeptide repeats. (A) The lytic cycle was induced in the stable HuARLT.BAC16.KSHV.WT and HuARLT.BAC16.KSHV.ΔvIRF2 cells with 12.5% tissue culture supernatant containing RTA-expressing baculovirus and 1.67 mM SB. At the indicated time points after induction the cells were lysed and the expression of the IFIT proteins 1, 2 and 3 as well as vIRF2 and K-bZIP was analyzed by WB. (B) Empty HuARLT or HuARLT.rKSHV.219 cells were transduced with a lentivirus expressing either the control GFP or the vIRF2 protein. 24 h after the transduction, reactivation was induced by 12.5% tissue culture supernatant containing RTA-expressing baculovirus and 1.67 mM SB and 48 h later cells were lysed and protein expression was analyzed by WB. (C) HuARLT cells were infected with either the KSHV.WT virus or the KSHV.ΔvIRF2 virus at a MOI of 5. The cells were lysed at the indicated time points and protein expression was analyzed by WB. IFIT protein levels were quantified to β-actin levels by using the Image studio software and are indicated below the blots setting the levels at 24 h post infection in WT-infected cells to 1.

To confirm the microarray results we induced the lytic cycle in KSHV.WT- and KSHV.ΔvIRF2-infected HuARLT cells and analyzed the IFIT protein expression by western blot. Consistent with the microarray data, we could detect a strong induction for all three IFIT proteins in the KSHV WT-infected cells already 24 h after reactivation. This increased IFIT expression was not detected in the KSHV.ΔvIRF2-infected cell line, in which the IFIT protein levels remained the same as in the absence of lytic induction (Fig 7A). As noted before (Fig 4C), the expression of the KSHV lytic K-bZIP protein was higher in KSHV.ΔvIRF2-infected compared to KSHV.WT-infected HuARLT cells (Fig 7A). In addition, we observed an increase in IFIT 1 and 3 protein levels upon KSHV reactivation in the PEL derived B cell line BC1 (S3A Fig). In this experiment, IFIT1 was not detectable in non-reactivated cells and its expression increases between 24 h and 48 h following reactivation. In contrast, IFIT3 showed a basal level of expression already in unstimulated cells, which increases at 72 h after reactivation (S3A Fig).

To investigate if vIRF2 causes the increased IFIT protein expression, we overexpressed vIRF2 by lentiviral transduction in either empty HuARLT or HuARLT.rKSHV.219 cells with or without reactivation. The vIRF2 overexpression in empty HuARLT cells resulted in a strong increase in the IFIT protein levels, which occurred independently of the reactivation cocktail (Fig 7B, left). The KSHV-infected endothelial cells, HuARLT.rKSHV.219 showed similar results, although the difference was not as strong as in the uninfected cells, probably because the basal vIRF2 expression in infected cells is already induced by lytic induction (Fig 7B, right). In this experiment we again noted the suppression of K-bZIP expression following overexpression of vIRF2 (Fig 7B, right). We also confirmed this vIRF2-mediated IFIT induction in uninfected primary endothelial cells by transducing human umbilical vein endothelial cells (HUVECs) with either the control or the vIRF2-expressing lentivirus. In this experiment, transduction of vIRF2 led to an increase in IFIT1 and 3 protein expression (S3B Fig). Furthermore, we analyzed PML and IFIT1 expression in immortalized endothelial cells (HuARLT) stably infected with KSHV.WT, KSHV.ΔvIRF2 or the HuARLT.BAC16.vIRF2 Stop mutants and their corresponding revertants following the activation of the lytic replication cycle (S3C Fig). We could observe a little variability between the expression of IFIT and PML in the different stop mutants which could be explained by their protein loading level (S3C Fig, see actin as a control). As already observed in the experiment shown in Fig 7A, IFIT1 expression was reduced in KSHV.ΔvIRF2-infected compared to KSHV.WT-infected cells (S3C Fig). In contrast, Stop#1- and Stop#2-infected cells, as well as their revertants, maintained the expression of IFIT1, indicating that the truncated vIRF2 proteins expressed by these mutants (Fig 6A) are sufficient to induce IFIT1 (S3C Fig).

As we could show that vIRF2 expression leads to an increased expression of the IFIT proteins, we wondered if this might correlate with the ability of vIRF2 to regulate early lytic viral protein expression during *de novo* infection. HuARLT cells were infected with either the KSHV.WT or the KSHV.ΔvIRF2 virus and IFIT protein expression was analyzed by western blot. As illustrated in Fig 7C, KSHV.WT strongly activates IFIT1-3 protein expression already 24 h after infection. In contrast, infection with the KSHV.ΔvIRF2 virus resulted in an attenuated, but still noticeable, induction of all three IFIT proteins. As noted before (Fig 5B), infection with KSHV.ΔvIRF2 led to an increased expression of the lytic proteins ORF45 and K-bZIP, whose expression correlated inversely with that of IFIT1-3 (Fig 7C).

These results suggest that vIRF2 activates IFIT1-3 expression on its own and in the context of KSHV infection.

### IFITs inhibit KSHV lytic protein expression in endothelial cells

**Fig 8:**
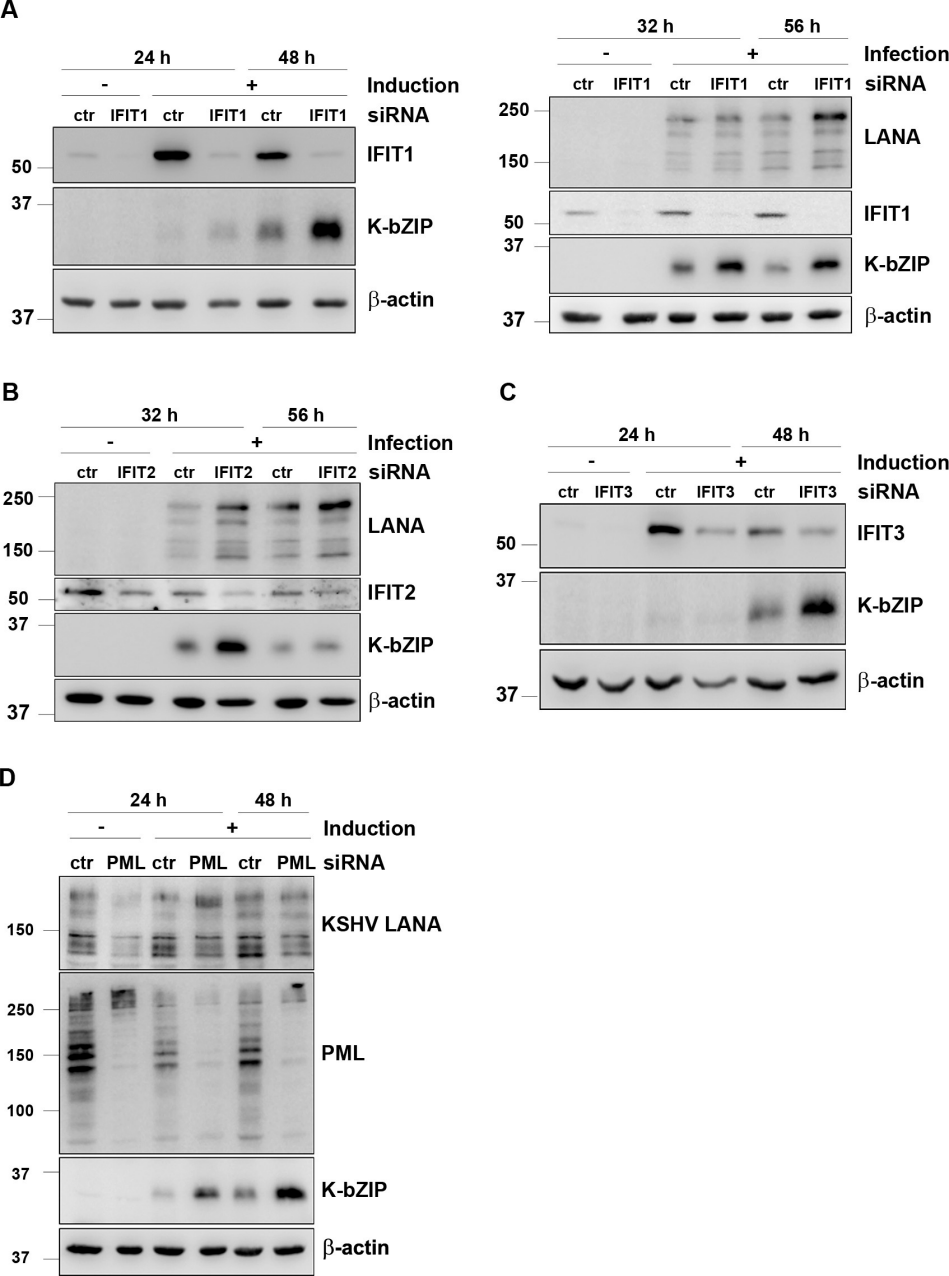
IFIT proteins and PML suppress KSHV lytic gene expression. (**A**) HuARLT.rKSHV.219 (left) or HuARLT (right) cells were microporated with a pool of four different siRNAs targeting IFIT1. 24 h later either the lytic cycle was induced with 10% tissue culture supernatant containing RTA-expressing baculovirus and 1.67 mM SB (left) or HuARLT cells were infected with rKSHV.219 at an MOI of 5 (right). (**B**) HuARLT cells were microporated with a pool of four different siRNAs targeting IFIT2. 24 h later cells were infected with rKSHV.219 at an MOI of 5. (**C**) HuARLT.rKSHV.219 cells were microporated with a pool of three different siRNAs targeting IFIT3. 24 h later the lytic cycle was induced with 10% tissue culture supernatant containing RTA-expressing baculovirus and 1.67 mM SB. All cells were lysed at the indicated time points and protein expression was analyzed by WB. (**D**) HuARLT.rKSHV.219 cells were microporated with a pool of three different siRNAs targeting all PML isoforms and 24 h later the lytic cycle was induced with 10% tissue culture supernatant containing RTA-expressing baculovirus and 1.67 mM SB. The cells were lysed at the indicated time points and analyzed by WB.

To understand the connection between the vIRF2-induced IFIT expression and the ability of vIRF2 to inhibit early lytic protein expression, we investigated if the IFIT proteins interfere with KSHV protein expression. We silenced individual IFITs by siRNA and measured the impact on lytic protein expression during reactivation as well as in newly infected cells. Silencing IFIT1 during KSHV lytic reactivation (Fig 8A, left) as well as during *de novo* infection (Fig 8A, right) increased the expression of the KSHV early lytic protein K-bZIP. IFIT2 knockdown affected K-bZIP expression only during KSHV *de novo* infection (Fig 8B and S4A Fig) and silencing of IFIT3 increased K-bZIP expression only during lytic reactivation (Fig 8C and S4B Fig). Notwithstanding the possibility that some of our siRNAs might have induced off-target effects, the ensemble of our findings suggest that all three IFIT proteins, but especially IFIT1, are able to restrict KSHV lytic protein expression during reactivation and/or *de novo* infection.

We also investigated if PML has a similar restrictive effect on KSHV lytic protein expression by silencing PML expression with siRNA during lytic reactivation in the immortalized endothelial cell line HuARLT.rKSHV.219. We found that a transient PML knockdown increases KSHV lytic protein expression, as shown for the early lytic protein K-bZIP (Fig 8D). Interestingly, we could observe this effect for PML only during lytic reactivation and not during a *de novo* infection (S4C Fig) which is in accordance with previously reported findings [74].

## Discussion

Among herpesviruses, vIRFs have so far only been found in Old World primate γ2 herpesviruses [30–32]. A number of studies have shown that the KSHV vIRFs can inhibit IFN-signaling and modulate anti-apoptotic as well as cell proliferation pathways [39]. Only a few studies have addressed the role of the KSHV vIRF proteins during KSHV replication. One study showed that KSHV vIRF4 facilitates lytic replication by targeting the expression of cellular IRF4 and c-myc [90]. In addition, vIRF4 interacts with CSF/CBF1, a downstream effector of Notch signaling that is also targeted by KSHV RTA and LANA and that is required for efficient lytic reactivation [42, 91]. Furthermore, vIRF4 has been shown to cooperate with RTA in the activation of several lytic promoters [92]. In contrast, vIRF3 has been shown to suppress, and vIRF1 to promote, lytic replication by recruitment of USP7 [40] or the BH3-only pro-apoptotic Bcl2 family member Bim [93]. However, in apparent contrast, vIRF3 degrades PML NBs in transfected cells, as reported before [44, 45] and shown in Fig 2.

Our investigation was prompted by the observation that infection of primary endothelial cells with KSHV does not result in the disruption of PML NBs, in contrast to infection with HCMV (Fig 1A and 1B) and to what has been noted for many other herpesviruses [46–56]. We noted an increase in the number of PML NBs following KSHV infection of endothelial cells (Fig 1A and 1B), reminiscent of an IFN-induced effect. Others have made a similar observation by showing IFN-induced PML transcription [74] and the increased expression of IFN-induced cellular genes following KSHV infection has been noted by several groups [73–75, 94]. In view of the ability of vIRF3 to degrade PML NBs [44, 45] and of several vIRFs to modulate IFN-signaling, we next compared the impact of all four KSHV vIRFs on PML expression and PML NBs. We found that vIRF2 promotes PML NB formation and increased PML protein levels, while vIRF1 and vIRF4 had no effect (Fig 2).

We next investigated the expression of the vIRF2 protein during KSHV latency and lytic replication in different cell lines with the help of two newly generated monoclonal antibodies that recognized the same repeated epitope located in an internal repeat region of vIRF2 (Fig 3B, S1 Fig). Our findings are consistent with previous studies that could detect increased vIRF2 mRNA levels after lytic cycle induction of different PEL as well as endothelial cells [26, 65, 67, 68]. In addition, our results suggest that several vIRF2 protein forms exist (Fig 3C and 3D). Some of them likely result from translational initiation at internal start codons, as they are produced by a KSHV mutant that had two stop codons inserted immediately after the predicted start codon for the vIRF2 ORF in exon K11.1 (Fig 6, Stop#1). Similarly, the 75 kDa vIRF2 band detected in some KSHV positive cell lines (Fig 3C) could also result from internal translation initiation at the beginning of the K11 exon and correspond to a truncated protein expressed only by the K11 exon. This 75 kDa band is not detectable in the nuclear fraction shown in Fig 3D, possibly as a result of lacking the NLS, which is located at the C-terminal end of the K11.1 exon (Fig 3B). Since our monoclonal antibodies recognize an epitope in the K11 exon, we cannot address the question whether a small protein that is only expressed from ORF K11.1 exists [35, 67]. In KSHV-infected and reactivated cells, the full-length vIRF2 protein is found both in the cytoplasm and in the nucleus (Fig 3D), as predicted from previous transfection experiments with a vIRF2 expression vector [69].

Using transient knockdown experiments with different vIRF2 siRNAs, as well as a KSHV mutant lacking the vIRF2 gene, we could show that vIRF2 restricts the expression of early lytic KSHV proteins both after reactivation from latency (Fig 4) and following *de novo* infection (Fig 5). By generating different KSHV mutants with translational stop codons inserted at different positions in the vIRF2 sequence we could show that the N-terminal part of vIRF2 which contains the DBD as well as an NLS is sufficient to inhibit KSHV lytic gene expression in endothelial cells (Fig 6).

A comparison of the cellular transcriptome in KSHV.WT- and KSHV.ΔvIRF2-infected cells showed that, at protein levels found in KSHV-infected cells, vIRF2 is a transcriptional regulator of different cellular genes (Table 1). This is in line with the fact that it binds to different cellular promoter regions [36]. Among the cellular genes involved in host defense that are differentially expressed in KSHV.WT- vs. KSHV.ΔvIRF2-infected endothelial cells (Table 1), we could identify the IFN-induced proteins with tetratricopeptide repeats 1, 2 and 3 (IFIT1, 2 and 3), whose expression we could show to be directly or indirectly induced by vIRF2 (Fig 7) and which restrict KSHV lytic protein expression (Fig 8). This finding extends the list of IFIT-restricted viruses, which so far only include two DNA viruses, HPV and HCMV [83, 84, 89] to another herpesvirus, KSHV.

Together our observations suggest that vIRF2 restricts KSHV early lytic protein expression by promoting the expression of IFN-regulated cellular genes that act as antiviral restriction factors. IFIT1-3 and PML are examples of such IFN-induced cellular proteins with the ability to restrict KSHV (Fig 8), but not necessarily the only ones that could contribute to the ability of vIRF2 to dampen KSHV lytic protein expression. It would therefore appear that vIRF2 enables KSHV to harness an antiviral cellular response to dampen lytic replication and therefore to establish or maintain latency. A role for type I interferons in the maintenance of latency has been reported for cytomegalovirus [95, 96]. Similarly, IFN-α promotes latency establishment in sensory neurons of the alphaherpesviruses HSV and pseudorabies virus [97]. Epstein-Barr virus latent membrane protein 1 (LMP1) has been shown to prime latently EBV-infected cells for the production of endogenous IFN and the activation of ISGs [98, 99]. In the case of these three examples, the virus takes advantage of IFN produced by infected or neighboring cells, whereas in the case of KSHV, vIRF2 can mimic or induce an IFN response in the absence of exogenous type I interferons. KSHV infection is known to enhance the expression of several ISGs and KSHV vFLIP has previously been shown to contribute to this process [73–75, 94]. In addition, a cytoplasmic variant of KSHV LANA has been shown to promote lytic reactivation by antagonizing the cGAS-dependent activation of the IFN pathway [100] and other KSHV proteins can modulate the function of cGAS [101–103]. KSHV vIRF2 may thus provide KSHV with an additional mechanism to harness an antiviral cellular mechanism for the purpose of latent viral persistence.

## Materials and methods

### Ethics statement

The use of human umbilical cords was approved by the Hannover Medical School Ethics Committee and experiments were performed in agreement with the Declaration of Helsinki. Written informed consent was obtained from parents of umbilical cord donors.

### Antibodies

The primary antibodies for western blot and immunofluorescence analysis are listed below. The antibodies goat anti-Calnexin (sc-6465), goat anti-Lamin A/C (sc-6215), mouse anti-IFIT2 (sc-390724), mouse anti-IFIT3 (sc-393512), mouse anti-KSHV K-bZIP (sc-69797), mouse anti-KSHV ORF45 (sc-53883) and mouse anti-KSHV K8.1 (sc-65446) were purchased from Santa Cruz Biotechnology. The primary antibody mouse anti-β-actin (A2228) was purchased from Sigma Aldrich, the mouse anti-GFP (632381) from Clontech, the rabbit anti-IFIT1 (D2X9Z) from Cell Signaling Technologies, the rabbit anti-PML (A301-167A) from Bethyl Laboratories, the rabbit anti-Daxx (1094–1) from Epitomics and the rabbit anti-Sp100 (AB1380) from Chemicon. For the detection of KSHV LANA we used a rat monoclonal anti-LANA antibody [104], which we produced from the hybridoma cell line. To detect vIRF2 the newly produced monoclonal mouse antibody clones #30F9 and #31A2 (see below) were used for western blot as well as for immunofluorescence (IFA). We used the mouse anti-PML (PG-M3, sc-966) from Santa Cruz Biotechnology to visualize PML nuclear bodies. The antibody to HCMV pp72 (IE1) was purchased from PerkinElmer (NEA-9221).

The secondary antibodies for western blot and IFA are listed below. The HRP-conjugated secondary antibodies rabbit anti-mouse IgG (P0447) and goat anti-rabbit IgG (P0448) were purchased from Dako. The HRP-conjugated rabbit anti-rat IgG antibody (3050–05) was purchased from SouthernBiotech. The HRP-conjugated mouse IgGκ light chain binding protein (sc-516102) was purchased from Santa Cruz Biotechnology and the 800CW infrared dye labeled goat anti-mouse IgG (926–32210) from Odyssey. The secondary antibodies for IFA, the FITC-conjugated goat anti-mouse IgG (115-095-146) and the Lissamine Rhodamine (LRSC)-conjugated goat anti-mouse IgG (115-025-072) were purchased from Jackson ImmunoResearch.

### Cell culture

HEK-293 (CRL-1573, American Type Culture Collection, ATCC), stable HEK-293.BAC16.KSHV and HeLa (CCL-2, ATCC) cells were cultured in DMEM (Gibco) supplemented with 10% fetal bovine serum (FBS, Sigma). Primary human umbilical vein endothelial cells (HUVECs) were isolated from umbilical cords by collagenase digestion as described before [105] and cultured in EGM-2MV BulletKit from Lonza. HuARLT cells, a conditionally immortalized human endothelial cell line derived from HUVECs [106], expressing the doxycycline-inducible simian virus 40 (SV40) large T antigen (TAg) and a human telomerase reverse transcriptase (hTert) (kindly provided by Dagmar Wirth, Helmholtz Centre for Infection Research Braunschweig), stable HuARLT.BAC16.KSHV and the cell line HuARLT.rKSHV.219, HuARLT cells latently infected with rKSHV.219 [73] were cultured in EGM-2MV BulletKit from Lonza or in Microvascular Endothelial Cell Growth Medium enhanced from PeloBiotech supplemented with 1 μg/ml doxycycline. HuARLT.rKSHV.219 cells were in addition cultured with 5 μg/ml puromycin. The PEL-derived B cell lines BC1 (CRL-2230, ATCC), BC3 (CRL-2277, ATCC) and BCBL1 (ACC 683, German Collection of Microorganisms and Cell Culture, DSMZ) as well as the B cell lines BJAB (ACC 757, DSMZ) and BJAB.rKSHV.219 (BJAB cells latently infected with rKSHV.219) [107, 108] were cultured in RPMI 1640 (Gibco) supplemented with 20% FBS. The BJAB.rKSHV.219 cells were in addition cultured with 4.2 μg/ml puromycin. Additionally, all stable HEK-293.BAC16.KSHV and HuARLT.BAC16.KSHV cells were cultured in the presence of 100 μg/ml hygromycin B. The stable HEK-293.BAC16.KSHV cell lines were generated by transfection of HEK-293 cells with 2 μg BAC DNA of a Maxi preparation (Macherey-Nagel, NuceloBond BAC100) with Fugene 6 transfection reagent (Roche, 11 814 443 001) according to the manufacturer’s protocol at a ratio of 3:1. Three days after transfection the cells were transferred from a 6 well plate into 10 cm dishes and BAC16 containing cells were selected by adding 100 μg/ml hygromycin B. The stable HuARLT.BAC16.KSHV cell lines were established by infection of HuARLT cells (at a MOI of 2) with the respective BAC16-derived virus produced in HEK-293.BAC16.KSHV cells. KSHV positive cells were selected with 100 μg/ml hygromycin B starting three days after infection.

HEK-293 cells were treated with IFNα (Sigma, SRP4596) for 72 h. Insect SF9 cells were cultured in spinner flasks at a density between 0.5 and 2∙10^6^ cells in grace’s insect media (Gibco).

### Reactivation, virus production and infection

Lytic KSHV reactivation in HuARLT.rKSHV.219, HuARLT.BAC16.KSHV and HEK-293.BAC16.KSHV cell lines was induced by treating the cells with 1.67 mM sodium butyrate (SB) and 10–30% SF9 cell culture supernatant containing KSHV RTA-expressing baculovirus. BJAB.rKSHV.219 cells were induced by adding 2.5 μg/ml anti-human IgM [107, 108].

The recombinant KSHV.219 virus was produced from BJAB.rKSHV.219 cells which were induced for three days with 2.5 μg/ml anti-human IgM, while BAC16-derived KSHV or KSHV mutants were produced in HEK-293.BAC16 cells using 1.67 mM SB and 30% tissue culture supernatant containing RTA-expressing baculovirus for three days. All virus containing supernatants used for infection were centrifuged for 5 min at 2500 rpm at 4°C, filtered with a 0.45 μM filter and ultracentrifuged for 4–5 h at 15–18,000 rpm at 4° C. The pellets were resuspended in medium without FBS and stored at 4°C.

To determine the titer of virus stocks, a serial dilution of the virus was prepared and added to 3x10^4^ HEK-293 cells in a 96 well plate, which had been seeded the day before. The plate was centrifuged for 30 min at 32°C and 450xg. After 72 h the viral titers were calculated by counting the number of GFP positive HEK-293 cells.

HUVEC or HuARLT cells were infected with either rKSHV.219, BAC16.KSHV.-derived virus or HCMV at the desired multiplicity of infection (MOI) in the presence of 8 μl/ml polybrene. The plates were centrifuged at 32°C and 450xg for 30 min.

The KSHV RTA-expressing baculovirus, used to induce the KSHV lytic cycle, was produced in SF9 cells. The cells were cultured in spinner flasks to a maximum density of 2x10^6^ cells. SF9 cells were seeded at a final cell density of 0.5x10^6^ cells/ml and infected with a baculovirus stock from a previous production. After four days the cells were collected and centrifuged at 1000 rpm for 20 min. The supernatant was filtered with a 0.45 μm filter and directly used for lytic cycle induction.

### Transfection and plasmids

Transfection of cells with plasmid DNA was performed with Fugene 6 (Roche, 11 814 443 001) according to the manufacturer’s protocol at a ratio of 3:1. The vector pCDNA3.1(+) was used as a control (V79020, Invitrogen) and the pcDNA3.1(+).vIRF1.myc/His, pcDNA3.1(+).vIRF3.myc/His and pcDNA3.1(+).vIRF4/His were kindly provided by Frank Neipel (Erlangen, Germany). To generate a pcDNA3.1 vector expressing full length vIRF2, vIRF2 cDNA was amplified from the PEL derived cell line BC3 after induction of the lytic cycle with 1.25 mM sodium butyrate and 10% tissue culture supernatant containing RTA-expressing baculovirus. RNA isolation was performed using the RNeasy Mini Kit (74104, Qiagen) with an additional on-column DNase digestion. The vIRF2 RNA was reversed transcribed by the expand reverse transcriptase (11785826001, Roche) and the cDNA was then amplified using 5’-TATGGATCCATGCCTCGCTACACGGAGTCGG-3’ and TATTCTAGATTACAGATCCTCTTCTGAGATGAGTTTTTGTTCGTCTCTGTGGTAAAATGGGGC-3’ followed by cloning into the pcDNA3.1(+) vector.

### Lentivirus production and transduction of endothelial cells

To generate the vIRF2 lentiviral vector a T2A element was introduced into the pcDNA3.1(+).vIRF2.myc vector (see above) carrying the vIRF2 cDNA by amplifying the T2A element from a vFLIP vector [73] using the primers 5’-TATGCTAGCAGGGCTCCGGAGAGGGCCGGGGCTCTC-3’ and 5’-TATGGATCCAGGGGCCGGGGTTCTCCTCCACGT-3’. Thereafter the T2A and vIRF2.myc fused product was amplified with 5’-TTGCTGTACAAGGGCTCCGGAGAGGGCCGGGGCTCTC-3’ and 5’-TATGTCGACTTACAGATCCTCTTCTGAGATGAGTTTTTGTTCGTCTCTGTGGTAAAATGGGGC-3’. The fragment was then digested with respective restriction enzymes and ligated into the lentiviral vector pRRL.PPT.SF.GFP (kindly provided by Axel Schambach, Medical School Hannover), previously linearized with *BsrgI* and *SalI* enzymes, to generate the pRRL.PPT.SF.vIRF2.T2A.GFP vector.

For lentivirus production, HEK-293.T cells (5∙10^6^ cells/10 cm dish) seeded the day before were transfected with 10 μg of the pRRL.PPT.SF.vIRF2.T2A.GFP plasmid or the corresponding control vector pRRL.PPT.SF.GFP as well as the helper plasmids pMDLGg/p (6.5 μg), pRSV–REV (2.5 μg), and pMD2.G (3.5 μg) (kindly provided by Renata Stripecke, Medical School Hannover) using the calcium phosphate method. The supernatants containing the lentivirus were collected after 36 h and 48 h. To concentrate the virus stock, the supernatants were filtered with a 0.45 μm filter and ultracentrifuged over night at 10,000 rpm and 4°C. The next day, the pellet was resuspended in medium without FBS and virus aliquots were stored at -80°C.

HuARLT cells were transduced by adding the calculated amount of virus to the cells in the presence of 8 μl/ml polybrene and centrifugation at 32°C and 450xg for 30 min.

### GST-protein production, purification and generation of a vIRF2 antibody

To produce a vIRF2 protein encoded by the K11 exon, the K11 sequence was cloned by restriction enzyme digestion and ligation into a pGEX-6P-1.GST vector and a 6xHis tag was fused to the C-terminus using the primers 5’-CGGGATCCAGGGAGGCCGCCAGGAAACAG-3’ and 5’-CCGGAATTCTTAGTGGTGATGGTGATGATGGTCTCTGTGG-3’. To produce the GST fused vIRF2 protein or the GST control after transformation of *E*. *coli* Rosetta cells, the bacteria culture was incubated at 37°C at 220 rpm until it reached a density between OD_600_ 0.4–0.6. The protein production was induced by adding 1 mM isopropyl-b-D-thiogalactopyranoside (IPTG, I6758, Sigma) and incubated for 4 h at 30°C. The culture was centrifuged for 10 min at 5000 rpm and 4°C and the pellet was resuspended in 100 ml resuspension buffer (1x PBS + protease inhibitors mix: 1 mM Aprotinin, 10 μM Leupeptin, 100 μM Phenylmethylsulfonylfluorid (PMSF), 1.46 μM Pepstatin A, 1 mM Benzamidine HCl). The cells were sonicated on ice five times for 30 sec and completely lysed by adding 0.5% NP40. After centrifugation for 10 min at 14,000 rpm and 4°C, the supernatant was collected and centrifuged again under the same conditions. For pull down of GST fused proteins, 1 ml glutathione-sepharose 4 Fast Flow beads (17513202, GE Healthcare) were washed in washing buffer (1x PBS, 0.5% NP40, 5% Glycerol) and added to the lysate. After incubation over night at 4°C while shaking gently, beads were washed three times in washing buffer. To elute the proteins from the beads, 2.5 ml elution buffer (1x PBS, 0.5% NP40, 10% Glycerol, 60 mM Glutathione, protease inhibitors mix, pH 7.3) were added and incubated for 3 h at 4°C while gently mixing. Afterwards, the solution was centrifuged, the supernatant was collected and recentrifuged. The protein solution was either stored at -80°C after adding 10% glycerol or dialyzed over night in a Slide-A-Lyzer Dialysis cassette (66332, ThermoFisher Scientific, 3.500 MWCO, 0.5–3 ml capacity) in dialysis buffer (1x PBS, 0.5% Glycerol, 100 μM PMSF). The next day the solution was isolated from the dialysis cassette and concentrated by using Amicon Ultra centrifugal filter devices (UFC910024, Millipore, 100.000 MWCO). The protein was run on a Coomassie gel (0.1% Coomassie Brilliant Blue R250 (Serva)) and protein concentration was calculated by Bradford using Roti-Quant (K015.2, Roth).

To produce the monoclonal antibodies against vIRF2/K11 C57BL/6J mice were immunized subcutaneously (s.c.) and intraperitoneally (i.p.) with a mixture of ~ 50 μg purified GST-tagged KSHV K11 protein, 5 nmol CpG (TIB MOLBIOL) and an equal volume of incomplete Freund's adjuvant (Sigma). After 6 weeks, a boost without adjuvant was given i.p. and s.c. 3 days before fusion. Fusion of the myeloma cell line P3X63-Ag8.653 (CRL-1580, ATCC) with the immune spleen cells was performed according to the standard procedure described by Koehler and Milstein [109]. After fusion, the cells were plated in 96 well plates using RPMI 1640 with 20% fetal calf serum (FCS), 1% penicillin/streptomycin, 1% glutamine, 1% sodium pyruvate, 1% non-essential amino acids, 2% HCS (Capricorn) and 1% HT supplement (Thermo Fisher). After 10 days, hybridoma supernatants were tested in an ELISA on plates coated with vIRF2/K11 protein (4 μg/ml). After blocking with 1x PBS/2% FCS, hybridoma supernatants were added for 30 min. After one wash with 1x PBS, bound antibodies were detected with a cocktail of HRP-conjugated mAbs against the four mouse IgG isotypes. HRP was visualized with ready to use TMB (1-StepTM Ultra TMB-ELISA, Thermo Fisher) and the absorbance was measured at 650 nm with a microplate reader (Tecan). The hybridoma cells of vIRF2/K11-reactive supernatants were cloned at least twice by limiting dilution. Experiments in this study were performed with hybridoma culture supernatant of vIRF2/K11 clone #30F9 and #31A2 (mouse IgG2b/κ) at a dilution of 1:10 in PBS-T.

### Antibody epitope mapping

The synthesis of the peptide array (SPOT synthesis) was carried out with an Intavis MultiPep automated SPOT array synthesizer (Intavis Bioanalytical Instruments, Cologne, Germany) on an amino-PEG functionalized SPOT synthesis paper membrane (AIMS Scientific Products, Berlin, Germany) with a size of 9x13 cm based on a published procedure [110]. The entire original protein sequence of K11 was divided into 169 overlapping peptides with a length of 15 amino acids and a shift of three amino acids for consecutive sequences. On each peptide position, ß-alanine was first coupled to the paper by using Fmoc-ßAla-OPfp (0.3 M) and HOBt (0.3 M) in NMP, to which 10 μl/ml diisopropylcarbodiimide was added. The activated amino acid was added at 0.2 μl to each position, reaction time was 45 min. Afterwards the free amino groups on the membrane were acetylated for 1 h with 2% acetic acid anhydride in DMF (capping solution). The Fmoc group from the ß-alanine was cleaved by an 8 min treatment with 20% piperidine in DMF. After washing with DMF, the SPOT peptide positions were stained with bromophenol blue (2% of the ethanol stock solution in DMF), followed by washing of the membrane with ethanol and drying. The corner positions of the array were marked with a pencil for a later identification of the peptide positions. The peptide sequences were assembled by utilizing Fmoc amino acid derivatives (0.2 M in NMP) with preactivation for 30 min by equimolar amounts of diisopropylcarbodiimide and hydroxybenzotriazol. 3 μl/ml of a stock solution of 10 mg/ml bromophenol blue in ethanol were added to each amino acid solution, which allowed the monitoring of the progress of the coupling reactions by observing the colour change from blue to green. The activated amino acids were spotted three times to each position at 0.2 μl. After the third spotting the reaction was allowed to proceed for a further 30 min, thereafter the membrane was washed with DMF and capping was carried out for 7 min. After final assembly of the peptide chains and cleavage of the terminal Fmoc group, the free N-terminus was acetylated with the capping solution for 15 min.

The membranes with the completed arrays were washed with DMF, ethanol and dried. Side chain protection groups were cleaved by two consecutive 2 h treatments with trifluoroacetic acid containing 5% dichloromethane, 3% diisopropyl silane and 2% water. Afterwards, the membranes were washed with dichloromethane and ethanol and finally dried. The ready-to-use peptide arrays were sealed in plastic foil and stored at -20°C until usage.

To map the epitope of the two K11 antibody clones #30F9 and #31A2, the membranes were wetted with a few drops of ethanol and blocked in 5% milk in PBS-T buffer for 2 h at RT. The membranes were incubated with the antibody clones #30F9 or #31A2 (diluted 1:10 in 5% milk PBS-T) over night at 4°C gently mixing. After washing the membranes three times in PBS-T, they were incubated with the secondary goat anti-mouse IgG antibody labeled with the IRDye 800CW for 1 h at RT in the dark. The membranes were developed in a LI-COR Odyssey after washing three times in PBS-T.

### Cell lysis

Cells were lysed after washing in 1x PBS in 1x SDS lysis buffer (62.5 mM Tris-HCl pH 6.8, 2% (W/V) SDS, 10% (V/V) Glycerol, 50 mM DTT, bromophenol blue). To pellet down cell debris, the samples were centrifuged after lysis for 10 min at 13,000 rpm at 4°C. If necessary, cell lysates were sonicated for a few seconds before centrifugation. The protein concentrations were measured using the spectrophotometer NanoDrop1000 (Peqlab).

### SDS PAGE and immunoblot

Cleared cell lysates were boiled for 5 min at 95°C and loaded on 8–12% SDS polyacrylamide gels. As a protein marker the Precision Plus Protein All Blue Prestained Protein Standards (1610373, Biorad) was used. After SDS PAGE, the proteins were transferred on a nitrocellulose membrane (Premium 0.45 μm, 10600003, Amershan) for 70 min at 350 mA in transfer buffer. Depending on the antibody, unspecific binding was blocked by incubating the membrane in either 5% milk in PBS-T or TBS-T buffer or in 5% bovine serum albumin (BSA) in TBS-T buffer. The membranes were incubated with the primary antibody on a roller at 4°C over night or 1 h at RT. After three washing steps in the corresponding buffer, the membranes were incubated for 1 h at RT in the secondary *horseradish peroxidase* (HRP)-conjugated antibody. Primary and secondary antibodies used for western blot are listed above (section antibodies). To visualize the specific detection of proteins, the membranes were developed in a LAS-3000 Imager (Fujifilm) using either self-made enhanced chemiluminescence (ECL) solution 1 and 2, which were mixed in a ratio of 1:1, or the SuperSignal West Femto Maximun Sensitivity Substrate (34096, Thermo Scientific).

### Fractionation assay

To isolate nuclear and cytoplasmic cellular fractions, the NE-PER Nuclear and Cytoplasmic Extraction Reagents from ThermoScientific (78833) were used following the manufacturer’s protocol for a packed cell volume of 50 μl.

### Generation of BAC16.KSHV mutants

To insert stop codons into the vIRF2 gene in the KSHV genome, competent *E*. *coli* GS1783 cells with chromosomally encoded inducible Red- and I-SceI expression were prepared by inoculation of 50 ml LB medium with an overnight culture (1:25) and incubated for 3 h at 32°C. The culture was shaking at 42°C for 15 min, followed by an incubation of 20 min while shaking in an ice bath. The bacteria were centrifuged for 10 min at 4,000 rpm at 4°C and the pellet was washed twice with sterile H_2_O and once with sterile ice cold 10% glycerol solution. The final pellet was resuspended in 600 μl 10% glycerol, aliquoted at 50 μl and the bacteria were either used directly or stored at -80°C.

In the first recombination step of the *En passant* mutagenesis, first described by Tischer et al. [111], the PCR amplicon of a kanamycin resistance gene from the pOri6K.I-SceI vector (kindly provided by Martin Messerle, Medical School Hannover) with an integrated I-SceI cleavage site with homologous flanking sequences carrying the mutation was inserted in competent *E*. *coli* GS1783 by electroporation (by using 0.2 cm Gene Pulser cuvettes (Bio-Rad) at 2.5 kV, 25 μF and 200 Ohm). The primers used for the different mutants are listed in the supplement (S1 Table). For DNA amplification the Phusion high-fidelity polymerase system from NEB (M0530L) was used. To amplify the kanamycin cassette for the Tischer mutagenesis, 0.2 μM of the forward primer were directly added to a 25 μl reaction whereas the same amount of the reverse primer was added after 17 PCR cycles in a Veriti 96 well thermal cycler from Applied Biosystems. The electroporated bacteria were selected on kanamycin and chloramphenicol and resistant clones were checked with restriction analysis.

The second recombination step aims to remove the kanamycin resistance again such as to leave only the introduced mutations. LB medium with chloramphenicol only was inoculated with of an overnight culture (1:50) and incubated for 3 h at 32°C in a shaker at 220 rpm. After adding 1% L-arabinose to induce I-SceI expression, the culture was incubated for 1 h at 32°C and transferred afterwards into a 42°C waterbath shaker for 25 min. After the heat shock, the culture was returned to 32°C for 3 h. A negative selection process revealed final clones which showed kanamycin sensitivity.

The BAC16.KSHV.ΔvIRF2 mutant was generated differently by using a galK/Kan*-*based selection in *E*. coli SW102 [112] essentially as previously described [113]. Briefly, a PCR amplified galK/Kan cassette, carrying 50 bp homologous regions flanking the vIRF2 gene (primers see S1 Table) was electroporated into *E*. *coli* SW102 carrying the BAC16.KSHV.WT (by using a 0.1 cm cuvette (BioRad) at 25 μF, 1.75 kV and 200 Ω). The electroporated bacteria were selected on McConkey plates with kanamycin and chloramphenicol.

The integrity of all mutants, and the presence of introduced mutations, was confirmed by restriction analysis and final clones were verified by sequencing the entire KSHV BAC by Next Generation Sequencing. Briefly, purified BAC DNA obtained from a maxi preparation was sheared by sonication. To avoid bias by over-amplification, library preparation was performed using the KAPA real-time library preparation kit (KAPA Biosystems, Wilmington, MA, USA) with a limited number of PCR cycles. Quality controlled libraries were sequenced on a MiSeq (Illumina) using reagent kit v3 to generate 2 x 300 base paired-end reads. Reads were mapped to the KSHV BAC16 parental strain and variants were identified by using the low frequency variant detector function in CLC genomics Workbench v9. All the KSHV BAC16 mutants used in this study showed only the mutations introduced by mutagenesis and did not contain any additional changes in their genomic sequence.

### siRNA transfection

Custom designed siRNAs for vIRF2 and for PML [114] were purchased from Thermo Scientific Dharmacon (vIRF2 (1): CGGAAUGGCUCACGGACUU; vIRF2 (2): UUUCGCUGUCACUCGAUUCUU; vIRF2 (3): UUCUUCGCGAUGCAUUUCCUU; PML: AGAUGCAGCUGUAUCCAAGUU [114]). For IFIT1 and IFIT2 a pool of four siRNAs for each target gene was purchased from Thermo Scientific Dharmacon (IFIT1: M-019616-01-0005; IFIT2: M-012582-01-0005). For IFIT3 a pool of three siRNAs was purchased from Santa Cruz (sc-75326). Upon delivery, siRNA pellets were resuspended in nuclease free water (P1193, Promega) following the manufacturer’s protocol (Thermo Scientific Dharmacon) to a final concentration of 100 μM. The siRNA was aliquoted and stored at -80°C. A non-targeting siRNA pool was used as a negative control (Pool 2 D-001206-14-20, Thermo Scientific Dharmacon).

For transfection of siRNA into HuARLT.rKSHV.219 or HUVECs the Neon Transfection System (ThermoFisher, MPK5000) was used. The transfection of 1x10^5^ cells with 150 pmol siRNA was performed using three single pulses of 1350 V each for 30 msec.

### Quantitative Real time PCR (qPCR)

Total RNA was isolated by using the RNeasy Mini Kit (74104, Qiagen) with an additional on-column DNase digestion.

For qPCR, cDNA was synthesized using the expand reverse transcriptase (11785826001, Roche) according to manufacturer’s instructions. Briefly, 1 μg of total RNA and 200 pmol of (dT) primer (MWG-Operon) and PCR grade H_2_O (Qiagen) was mixed in a 11.5 μl reaction. The mixture was incubated at 65°C for 10 min in a thermocycler, immediately cooled on ice and afterwards the following components were added: 1x Reverse expand transcriptase buffer, 10 mM DTT, 1 mM of each dNTP, 20 U of recombinant RNAsin (Promega), and 50 U recombinant expand reverse transcriptase to make a total volume of 20 μl. The mixture was then incubated at 43°C for 1 h and the synthesized cDNA was used for subsequent qPCR. To quantify PML mRNA expression in HUVEC, qPCR amplification was performed in a 10 μl reaction volume using Taqman universal PCR master mix according to manufacturer’s recommendations (Applied Biosystems Cat.No 4364341). The following Taqman gene expression assays were used: PML- Hs 00231241; GAPDHHs 02758991 and qPCR was performed in a Stratagene MX3000P thermocycler using the following thermo profile: hold at 50°C for 2 min, followed by initial denaturation at 95°C for 20 sec, followed by 40 amplification cycles each at 95°C for 3 sec and 60°C for 20 sec period.

### Immunofluorescence assay

To detect PML NBs, HeLa cells were plated on glass coverslips (2x10^5^ cells per well of a 24 well plate) transfected with the vIRF constructs or the control vector 24 h later and 48 h later cells were washed in 1x PBS and fixed with 4% paraformaldehyde (PFA) for 20 min at RT. For immunofluorescence in HUVECs, cells were plated on glass coverslips (2x10^5^ cells per well of a 24 well plate) and infected the following day with rKSHV.219 (MOI 20) or HCMV (MOI 3). After fixation, the cover slips were washed three times with 1x PBS and cells were permeabilized in 0.2% triton/PBS for 10 min at RT.

Unspecific binding was blocked by incubating the cells in 0.5% BSA for 30 min at 37°C after additional washing for three times in 1x PBS. The primary antibody for PML was diluted 1:200 in 0.5% BSA/PBS and cells were incubated for 30 min at 37°C. After washing the coverslips three times in 1x PBS, they were incubated with the secondary antibodies diluted 1:200 in 0.5% BSA/PBS for 1 h at 37°C. During the incubation with the secondary antibody the DAPI staining was performed in parallel. After this, the cells were washed twice in 1x PBS and once in ddH_2_O and mounted on slides in 30 μl moviol supplemented with DABCO. The slides were dried overnight in the dark at RT and images were taken with a ZEISS AxioObserver microscope.

### Microarray analysis

The Microarray analysis was performed by the Research Core Unit Transcriptomics (RCUT) of Hannover Medical School.

The Microarray utilized in this study represents a refined version of the Whole Human Genome Oligo Microarray 4x44K v2 (Design ID 026652, Agilent Technologies), called ‘054261On1M’ (Design ID 066335) developed at the Research Core Unit Transcriptomics (RCUT) of Hannover Medical School. Microarray design was created at Agilent’s eArray portal using a 1x1M design format for mRNA expression as template. All non-control probes of design ID 026652 have been printed five times within a region comprising a total of 181560 Features (170 columns x 1068 rows). Four of such regions were placed within one 1M region giving rise to four microarray fields per slide to be hybridized individually (Customer Specified Feature Layout). Control probes required for proper Feature Extraction software operation were determined and placed automatically by eArray using recommended default settings.

Synthesis of cRNA was performed with the ‘Quick Amp Labeling kit, no color’ (#5190–0447, Agilent Technologies), except that the NTP-mix (to be used in the T7 reaction) was exchanged for a mix composed of 15 mM of each ATP, CTP, GTP, 11.25 mM of UTP, and 3.75 mM of aaUTP (final concentration of each nucleotide was 1.875 mM). The labeling of aaUTP-cRNA was performed by use of the Amino Allyl MessageAmp II Kit (#AM1753; Life Technologies) and Alexa Fluor 555 Reactive Dye (#A32756; LifeTechnologies).

cRNA fragmentation, hybridization and washing steps were carried-out as recommended in the ‘One-Color Microarray-Based Gene Expression Analysis Protocol V5.7’ (Agilent), except that 1300 ng of each labelled cRNA population were used for hybridization.

Slides were scanned on the Agilent Micro Array Scanner G2565CA (pixel resolution 3 μm, bit depth 20).

Data extraction and processing of raw fluorescence intensity values were performed with the ‘Feature Extraction Software V10.7.3.1’ using the extraction protocol file ‘GE1_107_Sep09.xml’, except that ‘Multiplicative detrending’ algorithm was inactivated.

## Supporting information

S1 Fig Epitope mapping for two monoclonal vIRF2 antibodies by a peptide array of overlapping K11 peptides. (**A**) Schematic diagram of the GST-K11-6xHis expression construct compared to the entire vIRF2 gene. The vIRF2 gene consists of one intron and two exons, K11.1 and K11. For GST-protein production and purification, the K11 exon was fused to GST at the N-terminus and to a 6xHis tag at the C-terminus. (**B**) Peptide arrays were performed to identify the epitopes of the monoclonal antibodies #30F9 and #31A2. In total 169 overlapping peptides (3 aa shifts, 15 aa long) covering the whole K11 sequence were spotted on membranes: seven rows à 24 peptide spots and one row with one spot. After incubating with the antibody clones #30F9 or #31A2 over night at 4°C and with the secondary goat anti-mouse IgG antibody labeled with the IRDye 800CW, the membranes were developed in a LI-COR Odyssey. (**C**) List of the sequences of the peptides relevant for the epitope mapping, amino acids constituting the epitope are marked in red.
(TIF)

S2 Fig KSHV vIRF2 does not restrict lytic gene expression during reactivation in epithelial cells. Stably infected HEK-293.BAC16.KSHV.WT and ΔvIRF2 cells were induced using 10% tissue culture supernatant containing RTA-expressing baculovirus and 1.67 mM SB. Protein expression was analyzed by WB after lysis of the cells at the indicated time points after lytic induction.
(TIF)

S3 Fig The vIRF2-dependent induction of IFIT protein expression in different cell lineages. (**A**) The lytic cycle in BC1 cells was induced with 100 ng/ml TPA, cells were lysed at the indicated time points after induction and protein expression was analyzed by WB. (**B**) HUVECs were transduced with either the control or the vIRF2 expressing lentiviral vector and 48 h after transduction cells were lysed and protein expression was analyzed by WB. (**C**) The different stable HuARLT.BAC16 cell lines carrying KSHV.WT, KSHV.ΔvIRF2, the four KSHV mutants with internal stop codons in the vIRF2 gene and their revertants were induced using 12.5% tissue culture supernatant containing RTA-expressing baculovirus and 1.67 mM SB for 72 h. Protein expression was analyzed by WB after lysis of the cells. Stop #1, aa7-8; Stop #2, aa323-324; Stop #3, aa386-387; Stop #4, aa460-461. Rev. #1, revertant to Stop #1; Rev. #2, revertant to Stop #2; Rev. #4, revertant to Stop #4.
(TIF)

S4 Fig IFIT2 does not restrict lytic gene expression during reactivation and IFIT3 and PML do not restrict lytic gene expression during de novo infection. (**A**) HuARLT.rKSHV.219 cells were microporated with a pool of four different siRNAs targeting IFIT2. 24 h later the lytic cycle was induced with 10% tissue culture supernatant containing RTA-expressing baculovirus and 1.67 mM SB. Cells were lysed at the indicated times and analyzed for K-bZIP expression. (**B, C**) HuARLT cells were microporated with a pool of three different siRNAs targeting IFIT3 (B) or PML (C). Twenty-four hours later cells were infected with rKSHV.219 at an MOI of 5. Cells were lysed at the indicated time points and protein expression was analyzed by WB.
(TIF)

S1 Table List of Primers and the corresponding sequences. (DOCX)
