# Supplementary figures and images for: Kaposi’s sarcoma-associated herpesvirus vIRF2 protein utilizes an IFN-dependent pathway to regulate viral early gene expression
Source: PLoS Pathog. 2019 May 6;15(5):e1007743. doi: 10.1371/journal.ppat.1007743 (PMC6522069; doi:10.1371/journal.ppat.1007743)

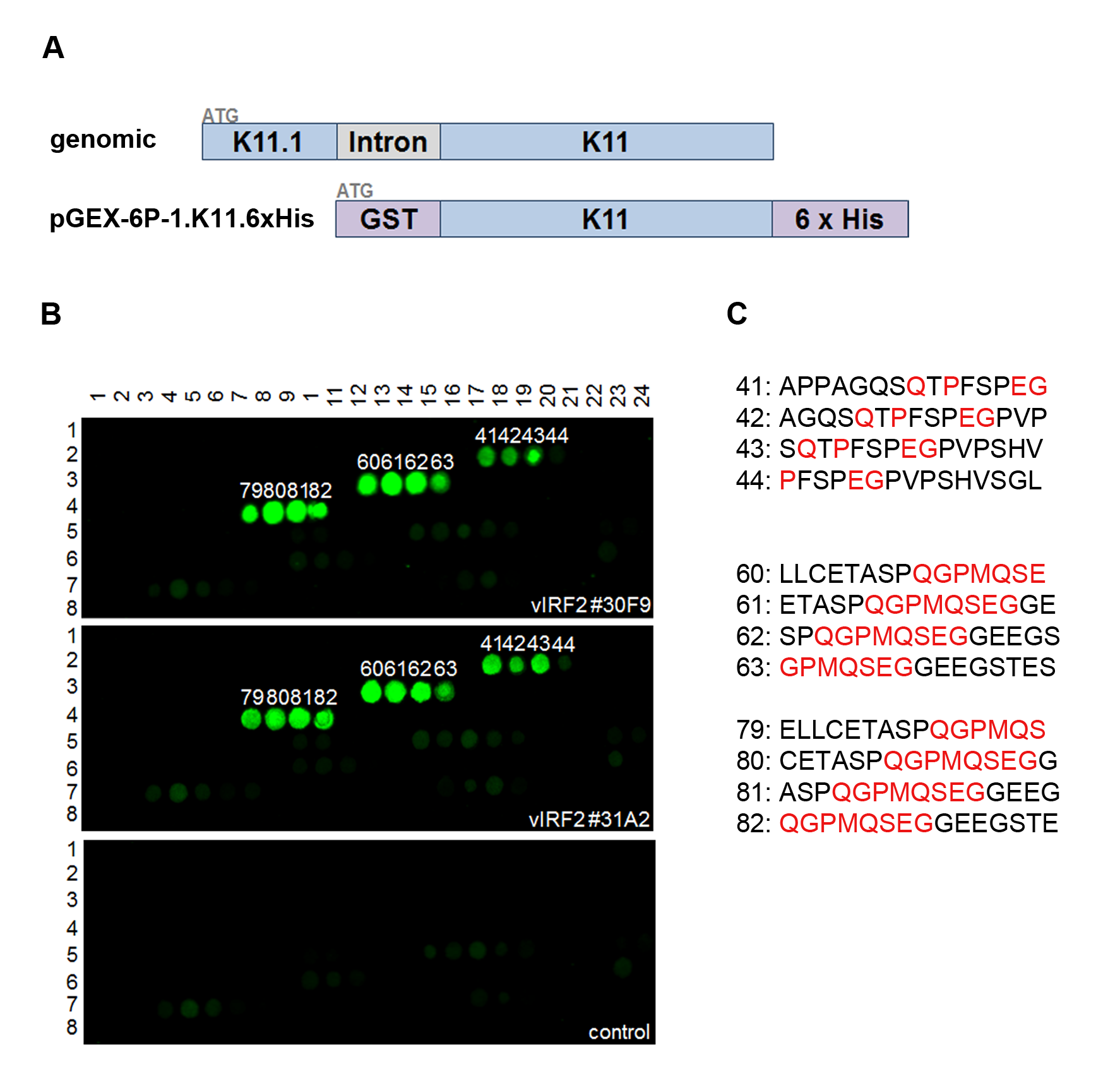

Supplement: S1 Fig — (A) Schematic diagram of the GST-K11-6xHis expression construct compared to the entire vIRF2 gene. The vIRF2 gene consists of one intron and two exons, K11.1 and K11. For GST-protein production and purification, the K11 exon was fused to GST at the N-terminus and to a 6xHis tag at the C-terminus. (B) Peptide arrays were performed to identify the epitopes of the monoclonal antibodies #30F9 and #31A2. In total 169 overlapping peptides (3 aa shifts, 15 aa long) covering the whole K11 sequence were spotted on membranes: seven rows à 24 peptide spots and one row with one spot. After incubating with the antibody clones #30F9 or #31A2 over night at 4°C and with the secondary goat anti-mouse IgG antibody labeled with the IRDye 800CW, the membranes were developed in a LI-COR Odyssey. (C) List of the sequences of the peptides relevant for the epitope mapping, amino acids constituting the epitope are marked in red. (TIF) [file ppat.1007743.s001.tif]

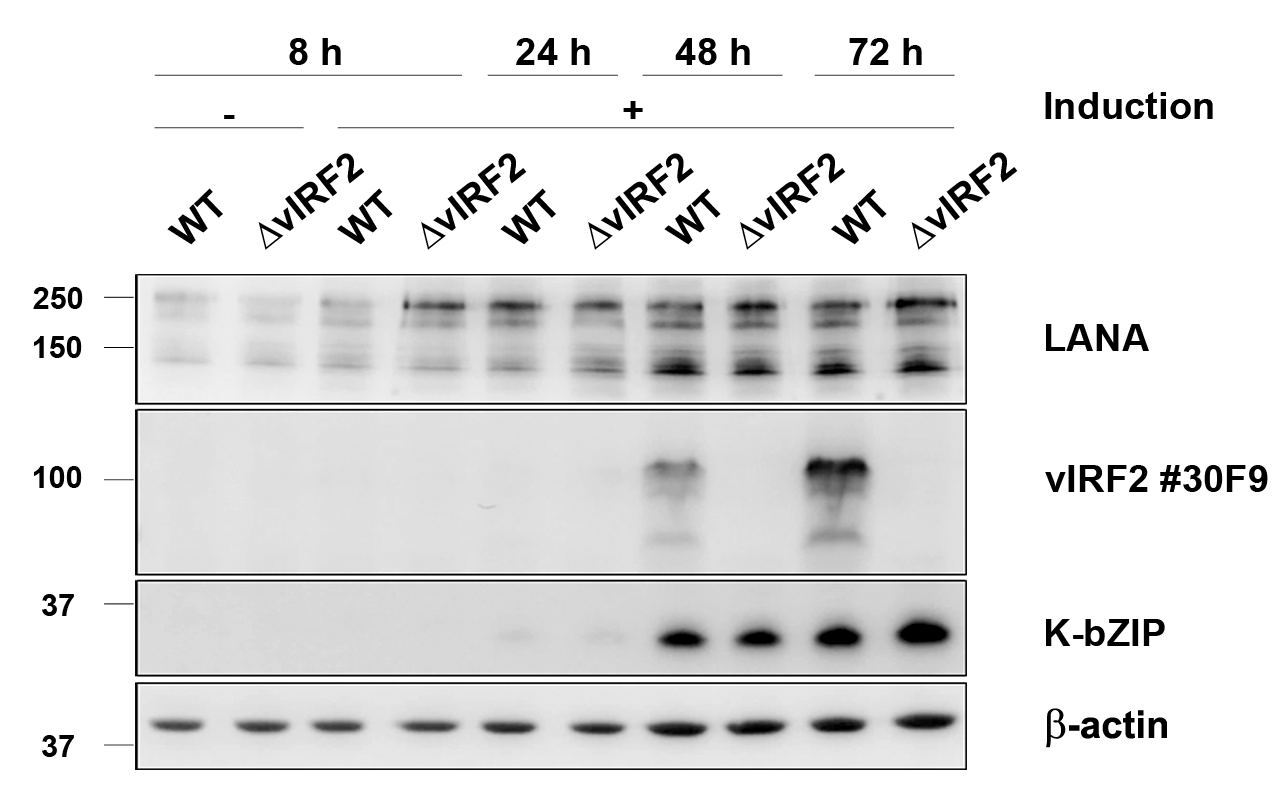

Supplement: S2 Fig — Stably infected HEK-293.BAC16.KSHV.WT and ΔvIRF2 cells were induced using 10% tissue culture supernatant containing RTA-expressing baculovirus and 1.67 mM SB. Protein expression was analyzed by WB after lysis of the cells at the indicated time points after lytic induction. (TIF) [file ppat.1007743.s002.tif]

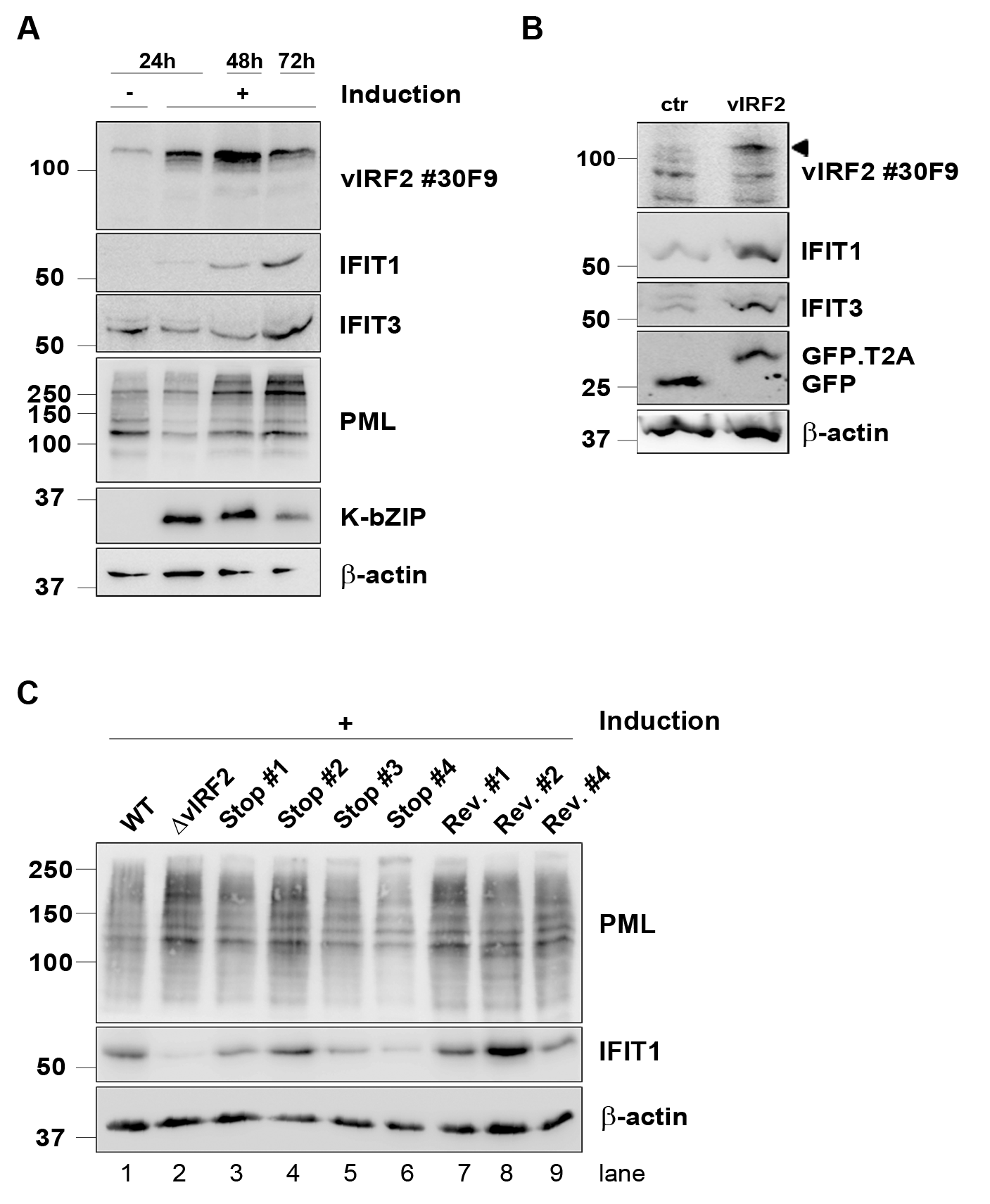

Supplement: S3 Fig — (A) The lytic cycle in BC1 cells was induced with 100 ng/ml TPA, cells were lysed at the indicated time points after induction and protein expression was analyzed by WB. (B) HUVECs were transduced with either the control or the vIRF2 expressing lentiviral vector and 48 h after transduction cells were lysed and protein expression was analyzed by WB. (C) The different stable HuARLT.BAC16 cell lines carrying KSHV.WT, KSHV.ΔvIRF2, the four KSHV mutants with internal stop codons in the vIRF2 gene and their revertants were induced using 12.5% tissue culture supernatant containing RTA-expressing baculovirus and 1.67 mM SB for 72 h. Protein expression was analyzed by WB after lysis of the cells. Stop #1, aa7-8; Stop #2, aa323-324; Stop #3, aa386-387; Stop #4, aa460-461. Rev. #1, revertant to Stop #1; Rev. #2, revertant to Stop #2; Rev. #4, revertant to Stop #4. (TIF) [file ppat.1007743.s003.tif]

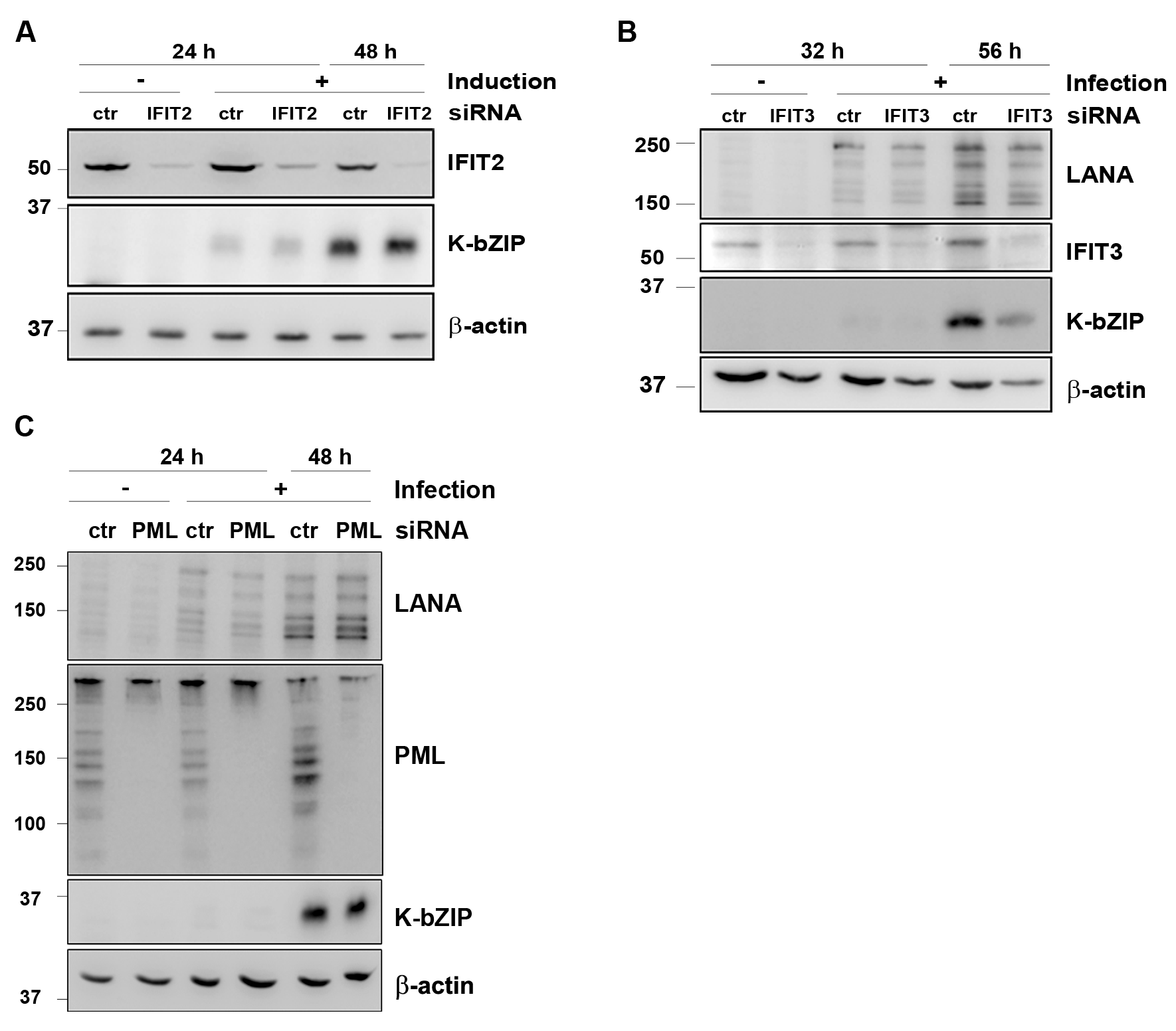

Supplement: S4 Fig — (A) HuARLT.rKSHV.219 cells were microporated with a pool of four different siRNAs targeting IFIT2. 24 h later the lytic cycle was induced with 10% tissue culture supernatant containing RTA-expressing baculovirus and 1.67 mM SB. Cells were lysed at the indicated times and analyzed for K-bZIP expression. (B, C) HuARLT cells were microporated with a pool of three different siRNAs targeting IFIT3 (B) or PML (C). Twenty-four hours later cells were infected with rKSHV.219 at an MOI of 5. Cells were lysed at the indicated time points and protein expression was analyzed by WB. (TIF) [file ppat.1007743.s004.tif]
