# Supplementary material for: Kaposi’s sarcoma-associated herpesvirus vIRF2 protein utilizes an IFN-dependent pathway to regulate viral early gene expression
Source: PLoS Pathog. 2019 May 6;15(5):e1007743. doi: 10.1371/journal.ppat.1007743 (PMC6522069; doi:10.1371/journal.ppat.1007743)
Supplement: S1 Table — (DOCX) [file ppat.1007743.s005.docx]

Table S1: List of primers and the corresponding sequences.

| **Primer Name** | **Nucleotide Sequence** |
| --- | --- |
| BAC16 Stop #1  aa7-8 Fwd | 5’-CCCACGGAAAAGGTGTTTTGTGTCGTGGCTTTTGCC  TAAAAAGATGCCTCGCTACACGGAGTAGTAATGGCTCACGGACTTTATTATGACGCATCGTGGCCGGATCTC-3’ |
| BAC16 Stop #1  aa7-8 Rev | 5’-GTCCACTGTCTAAAGCATCTATAATAAAGTCCGTGAG  CCATTACTACTCCGTGTAGCGAGGCATCTTTTTAGGCAAAAGCCACGACGTGACCACGTCGTGGAATGC-3’ |
| Rev BAC16 Stop #1  aa7-8 Fwd | 5’-CCCACGGAAAAGGTGTTTTGTGTCGTGGCTTTTGC  CTAAAAAGATGCCTCGCTACACGGAGTCGGAATGGCTCACGGACTTTATTATGACGCATCGTGGCCGGATCTC-3’ |
| Rev BAC16 Stop #1  aa7-8 Rev | 5’-TCCACTGTCTAAAGCATCTATAATAAAGTCCGTGAG  CCATTCCGACTCCGTGTAGCGAGGCATCTTTTTAGGCAAAAGCCACGACGTGACCACGTCGTGGAATGC-3’ |
| BAC16 Stop #2  aa323-324 Fwd | 5’-CTGGATGATTGCCTACCGATGGTGGATCACATTGA  GGGGTGTTTGTTAGATCTCTAGTGAGATGTTGGCCAGGAGCTTCCGACGCATCGTGGCCGGATCTC-3’ |
| BAC16 Stop #2  aa323-324 Rev | 5’-GTTCACCCAGGTCGCCTAAGTCAGGAAGCTCCTGG  CCAACATCTCACTAGAGATCTAACAAACACCCCTCAATGTGATCCACGTGACCACGTCGTGGAATGC-3’ |
| Rev BAC16 #2  Stop aa323-324 Fwd | 5’-CTGGATGATTGCCTACCGATGGTGGATCACATTGA  GGGGTGTTTGTTAGATCTCTTGTCAGATGTTGGCCAGGAGCTTCCGACGCATCGTGGCCGGATCTC-3’ |
| Rev BAC16 Stop #2  aa323-324 Rev | 5’-GTTCACCCAGGTCGCCTAAGTCAGGAAGCTCCTG  GCCAACATCTGACAAGAGATCTAACAAACACCCCTCAATGTGATCCACGTGACCACGTCGTGGAATGC-3’ |
| BAC16 Stop #3  aa386-387 Fwd | 5’-CGCCACGCATCCCCTTGAGAGTTCGGCACCTGGGG  CCTCTGTCATGGGTTCAGGCTAGTAGCTTCCTGACTTAGGCGACCTGGACGCATCGTGGCCGGATCTC-3’ |
| BAC16 Stop #3  aa386-387 Rev | 5’-CAGTTTCACACAGAAGTTCACTCAGGTCGCCTAAG  TCAGGAAGCTACTAGCCTGAACCCATGACAGAGGCCCCAGGTGCCGAACGTGACCACGTCGTGGAATGC-3’ |
| BAC16 Stop #4  aa460-461 Fwd | 5’-ATCTTTCCAAGCTTCCGACAATGTGGATGATTTTAT  TGATTGTATTCCACCGTAGTGACGTGATGACCGGGACGTCGAGGACGCATCGTGGCCGGATCTC-3’ |
| BAC16 Stop #4  aa460-461 Rev | 5’-TGGTCAGCTTTCTCTTGGTCCTCGACGTCCCGGTC  ATCACGTCACTACGGTGGAATACAATCAATAAAATCATCCACATTGTGACCACGTCGTGGAATGC-3’ |
| Rev BAC16 Stop #4 aa460-461 Fwd | 5’-CATCTTTCCAAGCTTCCGACAATGTGGATGATTTT  ATTGATTGTATTCCACCGTTGTGTCGTGATGACCGGGACGTCGAGGACGCATCGTGGCCGGATCTC-3’ |
| Rev BAC16 Stop #4  aa460-461 Rev | 5’-TGGTCAGCTTTCTCTTGGTCCTCGACGTCCCGGTC  ATCACGACACAACGGTGGAATACAATCAATAAAATCATCCACATTGTGACCACGTCGTGGAATGC-3’ |
| ΔvIRF2 Fwd | 5’-AAAGTCGCCCACGGAAAAGGTGTTTTGTGTCGTGG  CTTTTGCCTAAAAAGGAATTCCCTGTTGACAATTAATCATC-3’ |
| ΔvIRF2 Rev | 5’-AAGACAAAGGGAGGTACCCGGACAGAGTGACAA  GAAGACTTGTCAAAATTCTCAGCAAAAGTTCGATTTA-3’ |
